# O-GlcNAc transferase modulates the cellular endocytosis machinery by controlling the formation of clathrin-coated pits

**DOI:** 10.1016/j.jbc.2023.102963

**Published:** 2023-01-31

**Authors:** Sadia Rahmani, Hafsa Ahmed, Osemudiamen Ibazebo, Eden Fussner-Dupas, Warren W. Wakarchuk, Costin N. Antonescu

**Affiliations:** 1 Department of Chemistry and Biology, Toronto Metropolitan University, Toronto, Ontario, Canada; 2 Graduate Program in Molecular Science, Toronto Metropolitan University, Toronto, Ontario, Canada; 3 Department of Biological Sciences, University of Alberta, Edmonton, Alberta, Canada

**Keywords:** metabolism, O-GlcNAc transferase, clathrin mediated endocytosis, O-GlcNAc, AP-2 associated kinase, traffic, PICALM, EPSIN, hexosamine biosynthetic pathway, epidermal growth factor receptor, AAK1, AP2-associated kinase-1, AMPK, AMP-activated protein kinase, BSA, bovine serum albumin, CCP, clathrin-coated pit, CLS, clathrin-labeled structure, CME, clathrin-mediated endocytosis, DMEM, Dulbecco's modified Eagle's medium, EGFR, EGF Receptor, GBP, GFP-binding protein, O-GlcNAc, O-linked β-GlcNAc, sCLS, subthreshold clathrin-labeled structure, TfR, Transferrin Receptor, TIRF, total internal reflection fluorescence, TIRFM, TIRF microscopy, TMG, Thiamet G, WGA, wheat germ agglutinin

## Abstract

Clathrin-mediated endocytosis (CME) controls the internalization and function of a wide range of cell surface proteins. CME occurs by the assembly of clathrin and many other proteins on the inner leaflet of the plasma membrane into clathrin-coated pits (CCPs). These structures recruit specific cargo destined for internalization, generate membrane curvature, and in many cases undergo scission from the plasma membrane to yield intracellular vesicles. The diversity of functions of cell surface proteins controlled *via* internalization by CME may suggest that regulation of CCP formation could be effective to allow cellular adaptation under different contexts. Of interest is how cues derived from cellular metabolism may regulate CME, given the reciprocal role of CME in controlling cellular metabolism. The modification of proteins with O-linked β-GlcNAc (O-GlcNAc) is sensitive to nutrient availability and may allow cellular adaptation to different metabolic conditions. Here, we examined how the modification of proteins with O-GlcNAc may control CCP formation and thus CME. We used perturbation of key enzymes responsible for protein O-GlcNAc modification, as well as specific mutants of the endocytic regulator AAK1 predicted to be impaired for O-GlcNAc modification. We identify that CCP initiation and the assembly of clathrin and other proteins within CCPs are controlled by O-GlcNAc protein modification. This reveals a new dimension of regulation of CME and highlights the important reciprocal regulation of cellular metabolism and endocytosis.

The function of many proteins at the cell surface is regulated by clathrin-mediated endocytosis (CME), the principal mechanism of internalization of integral membrane proteins (1, 2, 3, 4, 5, 6). CME occurs as a result of the formation of clathrin-coated pits (CCPs) by the assembly of clathrin, the adaptor protein complex AP2, and many other proteins on the inner leaflet of the plasma membrane (3, 7, 8, 9, 10, 11). CCP formation is coupled to recruitment of specific integral membrane proteins (cargo), generation of membrane curvature, and eventual scission from the plasma membrane, yielding intracellular vesicles harboring protein cargo (1, 2).

The regulation of CCP dynamics by cues such as those derived from substrate stiffness (12), metabolism (13, 14), and growth factor stimulation (15) suggests that control of CME may contribute to broad regulation of cell surface protein function under various contexts. Understanding the cues that regulate CCP formation and CME is essential to understand how changes in endocytosis may contribute to cellular adaptation to various conditions.

The initiation of CCPs is dependent on the recruitment of the central adaptor protein AP2 to the plasma membrane, which then supports clathrin recruitment. AP2 membrane recruitment is enhanced by binding to the FEI complex comprised of FCHo, Intersectin, and Eps15 (16, 17). AP2 membrane binding is also enhanced by a conformational change in AP2 following binding to phosphatidylinositol-(4,5)-bisphosphate and specific sequence motifs on cargo proteins (5). The conformational change in AP2 is further stabilized by phosphorylation by AP2-associated kinase-1 (AAK1) or BMP2K on Thr156 of the μ2-subunit of AP2 (5, 18, 19, 20, 21, 22).

While some CCPs can lead to the formation of clathrin-coated vesicles, a large proportion of nascent CCPs undergo abortive turnover at the plasma membrane (5, 6, 21, 23). This indicates the existence of an endocytic checkpoint, in which the early stages of CCP formation undergo surveillance, resulting in either the further progression of suitable CCPs for vesicle formation or CCP disassembly without leading to vesicle formation. The GTPase dynamin2 (6, 24, 25) and AAK1-mediated phosphorylation of AP2 μ2 (21) may regulate abortive turnover of some CCPs. However, how AAK1 is regulated and how this regulation of AAK1 may thus control CCP assembly remain poorly understood.

The generation of membrane curvature within CCPs is required for the eventual generation of internalized coated vesicles. Membrane curvature within CCPs can be driven by several proteins, including Epsin and CALM, or BAR domain–containing proteins such as endophilin, amphiphysin, and SNX9 (1, 2, 3). CCPs may acquire curvature subsequent to the assembly of clathrin and other proteins at the plasma membrane (26, 27) or throughout the assembly of CCPs (28). Both mechanisms can be resolved in the same cell (29), suggesting that CCP assembly and curvature generation can be flexible (30). However, not all clathrin assemblies acquire curvature, and the assembly of so-called flat clathrin plaques may be triggered by growth factor receptor activation and may serve to control signaling outcome rather than mediate internalization (31).

The size of CCPs is also subject to regulation, which for example can occur by recruitment of certain cargo proteins such as low-density lipoprotein receptor as well as by the specific adaptors dab2 and ARH to CCPs (32). CCP size is also regulated by phosphatidylinositol-(4,5)-bisphosphate (33) and specific CCP accessory proteins such as CALM and NECAP (34, 35). Notably, CALM regulates CCP size and curvature, suggesting that generation of membrane curvature and CCP size may be linked (35). Hence, both CCP size and membrane curvature can be modulated to control CCP function, but which specific cellular signals control CCP size and/or curvature remains incompletely understood.

Signals derived from cell metabolism are emerging regulators of endocytic membrane traffic (13, 14, 36). For example, the metabolic sensor AMP-activated protein kinase (AMPK) is activated under conditions of nutrient limitation. AMPK activation controls the endocytosis of specific proteins such as the glucose transporters GLUT1 (37) and GLUT4 (38) and many others (13, 14). We previously found that many proteins including β1-integrin undergo changes in cell surface abundance upon AMPK activation (39). Collectively, these works suggest that metabolic signals may exert additional, still poorly appreciated effects on the regulation of endocytic membrane traffic.

A regulatory mechanism that has emerging roles in linking cell metabolism with regulation of various cellular process is the dynamic modification of nucleocytoplasmic proteins with 2-acetamido-2-deoxy-D-glucopyranose (GlcNAc) linked by a β-glycosidic bond to serine or threonine residues of proteins (O-GlcNAc) (40, 41, 42). The dynamic addition and removal of O-GlcNAc from proteins is regulated by two enzymes: a glycosyltransferase (uridine diphosphate-*N*-acetylglucosamine:polypeptide β-acetylglucosaminyltransferase; OGT) (43) and a glycoside hydrolase (O-GlcNAcase; OGA) (44). Deletion of OGT results in embryonic lethality, demonstrating the importance of this posttranslational modification (45).

O-GlcNAc cycling is thus controlled by a single glycosyltransferase, OGT, and has a single glycosidase, OGA, which can target as many as 8000 proteins for O-GlcNAc modification (45, 46). Importantly, O-GlcNAc protein modification uses UDP-GlcNAc as a substrate, a metabolite formed through the hexosamine biosynthetic pathway (47). Thus, the availability of specific nutrients such as glucose and glutamine modulates the extent of O-GlcNAc posttranslational modifications catalyzed by OGT and OGA, linking cell metabolism to control of a wide range of cellular processes (40, 41, 42).

O-GlcNAcylation impacts the function of target proteins in many ways specific to the protein being modified, which includes changes in protein half-life, enzyme activity, subcellular localization, and protein–protein interactions. Interestingly, O-GlcNAc modification of COPI and COPII components suggest the regulation of biosynthetic membrane traffic (48, 49, 50). In addition, the key regulator of CCP assembly and function, AAK1, may be subject to O-GlcNAc modification (51). However, how OGT and OGA, and in turn protein O-GlcNAcylation, may regulate CCP formation and CME is not known. Here, we examine how perturbations of OGT and OGA modulate the initiation, assembly, maturation, and scission of CCPs, and how dynamic protein O-GlcNAcylation of key CCP regulatory proteins such as AAK1 may impact this phenomenon.

## Results

### Protein O-GlcNAcylation regulates CCP dynamics

**Figure 1:**
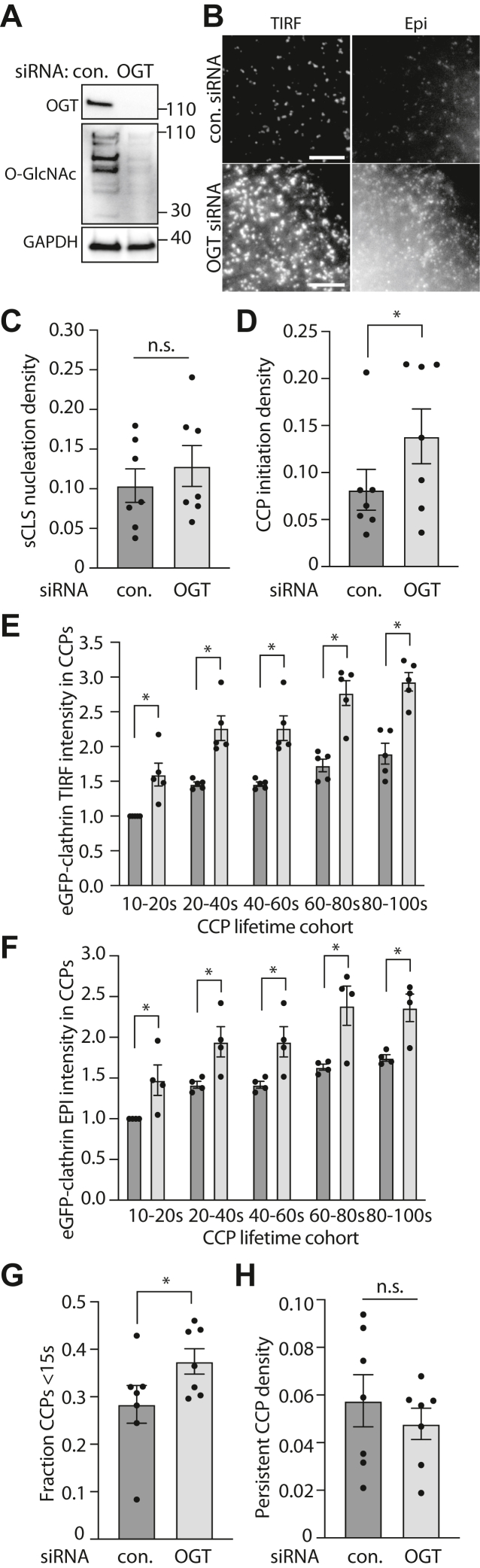
**OGT silencing impacts CCP initiation, size, and lifetimes.** ARPE-19 cells stably expressing eGFP-clathrin light chain were treated with siRNA to silence OGT or nontargeting (control) siRNA. *A*, shown are representative immunoblots of whole cell lysates probed with antibodies as indicated. All Western blot panels show approximate molecular weight in kDa. *B*–*H*, following transfection, cells were subjected to imaging with near-simultaneous time-lapse TIRF and epifluorescence microscopy imaging. Shown in (*B*) are representative images; scale bar represents 5 μm. These time-lapse image series were subjected to automated detection, tracking, and analysis of CLSs as described in Experimental procedures. Shown are the initiation rates of sCLSs (*C*) and bona fide CCPs (*D*). Also shown are the intensities of eGFP-clathrin in CCPs based on lifetime cohorts detected in TIRF (*E*) or epifluorescence (*F*) time lapse image series, as well as the fraction of CCP lifetimes <15 s (*G*) and the fraction of persistent CCPs (*H*). In each case (*C*–*H*), the data is presented as the mean (bar) ± SE from seven independent experiments, as well as individual values from each experiment (dots). The number of total sCLS trajectories, CCP trajectories, and cells (respectively) for each condition are as follows: control siRNA 9987, 8733, 45 and OGT siRNA 13015, 15220, 42. ∗*p* < 0.05. CCP, clathrin-coated pit; sCLS, subthreshold clathrin labeled structure; TIRF, total internal reflection fluorescence.

To examine how OGT and protein O-GlcNAcylation may regulate CCP formation and dynamics and thus CME, we used siRNA gene silencing. As expected, OGT silencing results in a robust reduction in both OGT expression as well as the levels of protein O-GlcNAc modification (Fig. 1*A*). We then used this in combination with the study of CCP dynamics using total internal reflection fluorescence (TIRF) microscopy (TIRFM) time-lapse imaging of cells expressing eGFP-tagged clathrin light chain (4, 5, 6). This approach has been previously used to reveal how various perturbations or other experimental conditions affect specific stages of CCP initiation, clathrin assembly, curvature generation, and CCP maturation (6). In TIRFM, the intensity of fluorophores and thus eGFP-clathrin objects declines with distance from the coverslip interface and therefore the intensity of eGFP-clathrin within CCPs decreases with acquisition of membrane curvature by CCPs. Hence, we performed imaging using near-simultaneous TIRF and widefield epifluorescence imaging modes (Fig. 1*B*), as the latter does not exhibit intensity changes based on nanoscale changes in CCP curvature (6). These image series are subject to automated image analysis involving detection, tracking, and analysis of diffraction-limited eGFP-clathrin objects using the TIRF channel for CCP detection.

This method can resolve each clathrin object as either a *bona fide* CCP or a subthreshold clathrin labeled structure (sCLS), as previously described (4, 5, 6). sCLSs are quantitatively and systematically distinguished from CCPs as they do not meet a minimum clathrin intensity threshold within the early stages of formation. As such, sCLSs likely represent stochastic assemblies of clathrin at the plasma membrane that fail to proceed to the formation of *bona fide* CCPs (4, 5, 6). Silencing OGT did not cause a statistically significant change in the rate of sCLS nucleation (Fig. 1*C*), but in contrast resulted in a significant increase in the rate of initiation of *bona fide* CCPs (Fig. 1*D*). This suggest that loss of protein O-GlcNAcylation permits a more efficient assembly of proteins such as clathrin and AP2 at the earliest stages of CCP formation.

An increased efficiency of recruitment of clathrin and other proteins into CCPs at the earliest stages of CCP formation would be expected to lead to a higher rate or amount of eGFP-clathrin recruited per CCP. To test this, we examined the levels of eGFP-clathrin recruited to CCPs. Silencing OGT resulted in a robust and significant increase in the levels of eGFP-clathrin in clathrin structures in TIRF image series (Fig. 1*E*). Since illumination of fluorophores decays exponentially with distance from the coverslip and thus the cell surface, an increase in eGFP-clathrin intensity in TIRF images could reflect a reduction of membrane curvature generation and/or an increased recruitment of eGFP-clathrin to each object. This would in turn result in no change or increased eGFP-clathrin intensity in corresponding structures in widefield epifluorescence image series, respectively. OGT silencing robustly and significantly increased eGFP-clathrin intensity in clathrin objects in widefield epifluorescence image series (Fig. 1*F*), suggesting that loss of OGT leads to enhanced recruitment of clathrin to CCPs and thus larger CCPs.

Following initiation, a subset of CCPs undergo abortive turnover, resulting in this subset of CCPs having shorter lifetimes than CCPs that lead to vesicle formation (5, 6, 21, 23). To determine if the increased rate of eGFP-clathrin recruitment into CCPs upon OGT silencing was associated with changes in the rate of abortive CCP turnover, we examined CCP lifetimes. OGT silencing significantly increased the fraction of CCPs with lifetimes <15 s (Fig. 1*G*) but did not change the proportion of CCPs that are persistent (present at the start and end of the time-lapse) (Fig. 1*H*). These results suggest that while OGT silencing leads to an increased rate of CCP initiation and of eGFP-clathrin recruitment into nascent CCPs resulting in larger CCPs, these CCPs may have a higher propensity for abortive turnover.

A separate siRNA sequence targeting OGT also achieved similar silencing of OGT and reduction of total cell modification of proteins with O-GlcNAc (Fig. S1*A*). Consistent with the results obtained by analysis of time-lapse TIRFM images showing that loss of OGT increased the size of CCPs, silencing of OGT using either siRNA sequence resulted in an increase in the level of eGFP-clathrin within clathrin structures in fixed cells, both in images obtained by TIRF (Fig. S1, *B* and *C*) and corresponding widefield epifluorescence (Fig. S1, *B* and *D*) microscopy. These results support the findings obtained in Figure 1 and suggest that the effects of silencing OGT that impact the size of clathrin structures are unlikely to be due to off target effects of a single siRNA sequence targeting OGT.

**Figure 2:**
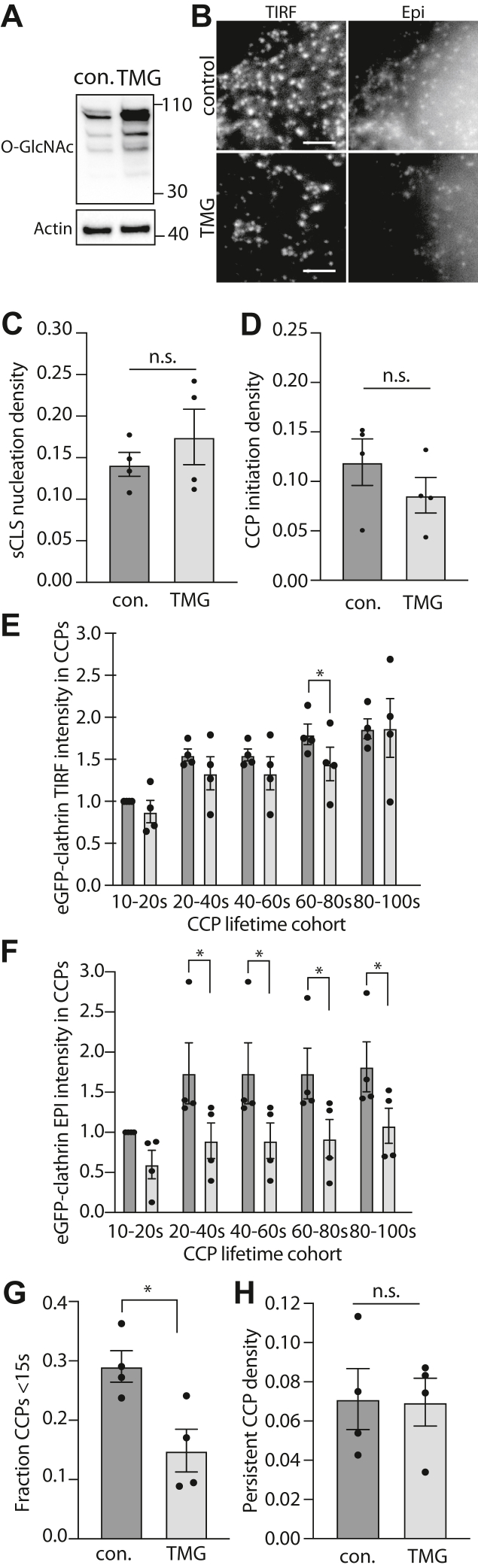
**Inhibition of OGA with Thiamet G treatment impacts CCP size and lifetimes.** ARPE-19 cells stably expressing eGFP-clathrin light chain were treated with 20 μM Thiamet G (TMG) or treated with vehicle control (con) for 4 h. *A*, shown are representative immunoblots of whole cell lysates probed with antibodies as indicated. All Western blot panels show approximate molecular weight in kDa. *B*–*H*, following transfection, cells were subjected to imaging with near-simultaneous time-lapse TIRF and epifluorescence microscopy imaging. Shown in (*B*) are representative images; scale bar represents 3 μm. These time-lapse image series were subjected to automated detection, tracking, and analysis of CLSs as described in Experimental procedures. Shown are the initiation rates of sCLSs (*C*) and bona fide CCPs (*D*). Also shown are the intensities of eGFP-clathrin in CCPs based on lifetime cohorts detected in TIRF (*E*) or epifluorescence (*F*) time lapse image series, as well as the fraction of CCP lifetimes <15 s (*G*) and the fraction of persistent CCPs (*H*). In each case (*C*–*H*), the data is presented as the mean (bar) ± SE from four independent experiments, as well as individual values from each experiment (dots). The number of total sCLS trajectories, CCP trajectories, and cells (respectively) for each condition are as follows: control 13063, 6479, 39 and TMG 10129, 3132, 21. ∗*p* < 0.05. CCP, clathrin-coated pit; sCLS, subthreshold clathrin labeled structure; TIRF, total internal reflection fluorescence.

Silencing OGT is an effective strategy to alter the levels of cellular protein O-GlcNAc modification, resulting in near complete loss of O-GlcNAcylation (Fig. 1*A*). We next sought to determine the impact of increasing cellular protein O-GlcNAcylation on CCP behavior. However, silencing OGA also causes loss of OGT, which results in only a modest change in cellular protein O-GlcNAc modification upon OGA silencing (52). In contrast, treatment of cells with Thiamet G (TMG), a small molecule inhibitor of OGA, leads to a robust increase in cellular protein O-GlcNAc modification (Fig. 2*A*). We thus examined how treatment with TMG impacts CCP dynamics as measured by near-simultaneous time-lapse imaging using TIRF and widefield epifluorescence microscopy (Fig. 2*B*) and image analysis. TMG treatment did not cause a statistically significant change in the rate of sCLS nucleation (Fig. 2*C*) or the rate of CCP formation (Fig. 2*D*).

Our experiments with OGT silencing revealed that O-GlcNAc modification may suppress the rate of recruitment of eGFP-clathrin to CCPs and thus modulate CCP size. Consistent with this, TMG treatment resulted in a modest decrease in eGFP-clathrin in TIRF image series for some CCPs (Fig. 2*E*) and a robust reduction in eGFP-clathrin per CCP in corresponding widefield epifluorescence image series (Fig. 2*F*). The decrease in eGFP-clathrin per CCP in epifluorescence images indicates that TMG treatment reduces the levels of eGFP-clathrin recruited per CCP, while the more modest effect on eGFP-clathrin intensity per CCP in TIRF images upon TMG treatment may also indicate that CCPs formed under these conditions do not exhibit full curvature generation.

Interestingly, TMG treatment also resulted in a robust reduction in the fraction of CCPs with lifetimes <15s (Fig. 2*G*) and did not impact the fraction of persistent CCPs (Fig. 2*H*), an observation complementary to the effect of OGT silencing. This suggests that TMG treatment reduces the abortive turnover of CCPs, favoring instead longer-lived productive CCPs. Collectively, this in turn suggests that cellular protein O-GlcNAc modification may modulate the rate of recruitment of proteins into nascent CCPs, such that protein O-GlcNAcylation suppresses the rate of assembly of proteins such as eGFP-clathrin into CCPs. Further, this suggests that loss of O-GlcNAcylation may lead to aberrant formation or growth of CCPs, which elicits an increase in abortive CCPs.

### OGT controls cargo recruitment to CCPs

**Figure 3:**
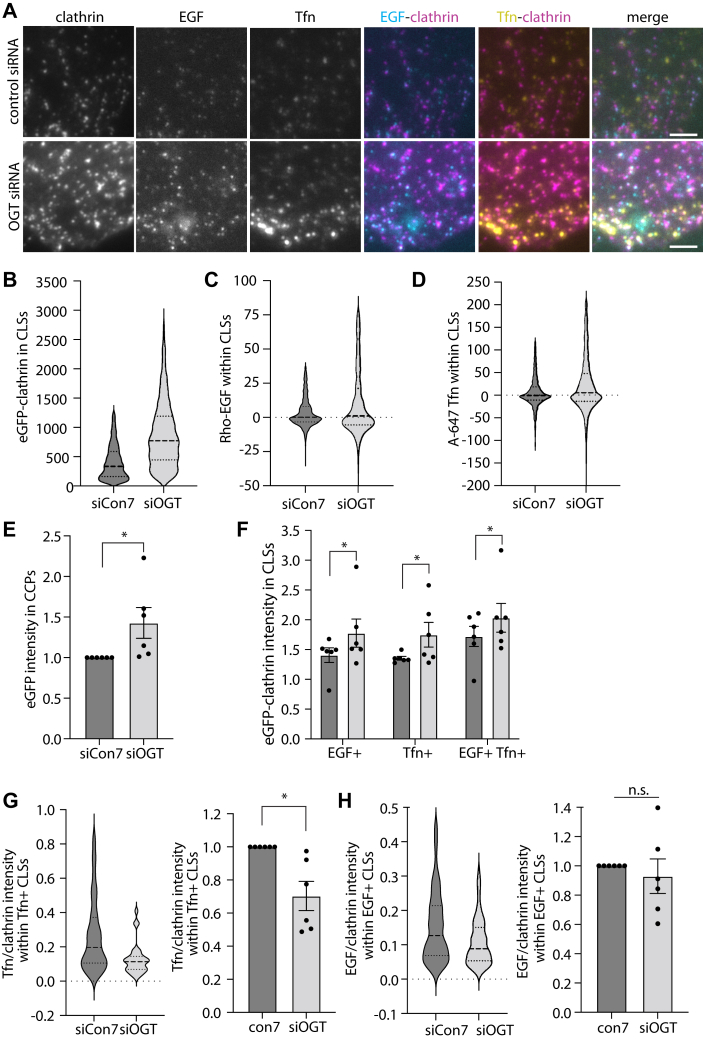
**OGT silencing impacts cargo recruitment to CCPs.** ARPE-19 cells stably expressing eGFP-clathrin light chain were treated with siRNA to silence OGT or nontargeting (control) siRNA. Then, cells were treated with 20 ng/ml rhodamine-EGF (rho-EGF) and 10 μg/ml A647-Tfn for 5 min, followed by fixation and imaging by TIRF and epifluorescence microscopy, with representative images shown in (*A*); scale bar represents 3 μm. CLSs were subjected to automated detection and analysis as described in Experimental procedures. Shown are the distributions as a violin plot of measurements of eGFP-clathrin (*B*), rhodamine-EGF (*C*), and A647-Tfn (*D*) in individual CLSs within a representative experiment, as well as the mean (bar) ± SE of eGFP-clathrin in CLSs from six independent experiments, as well as individual values from each experiment (dots). *F*–*H*, CLSs were sorted based on the presence of EGF or Tfn as described in Experimental procedures. *F*, shown is the mean (bar) ± SE of eGFP-clathrin in CLSs sorted by cargo type from six independent experiments, as well as individual values from each experiment (dots). Also shown is the ratio of A647/eGFP-clathrin (*G*) or rhodamine-EGF/eGFP-clathrin (*H*) intensities in CLSs, depicting the distribution of this ratio in a representative experiment as a violin plot (*left panels*), or the mean (bar) ± SE from six independent experiments, as well as individual values from each experiment (dots). The total number of CLSs quantified are as follows: control siRNA 18823 (total), 2826 (EGF+), 2828 (Tfn+); OGT siRNA 16227 (total), 2609 (EGF+), 2735 (Tfn+). ∗*p* < 0.05. CCP, clathrin-coated pit; TIRF, total internal reflection fluorescence.

The increase in CCP size upon silencing OGT may reflect changes in CCP assembly and composition that also impact the recruitment of specific cargo to CCPs. Given that some cargo such as Transferrin Receptor (TfR) and EGF Receptor (EGFR) are recruited to largely distinct CCPs and that CCPs harboring EGFR are uniquely regulated, such as by Ca2+ signaling (15), we next examined how OGT and cellular protein O-GlcNAcylation may uniquely impact recruitment of EGFR and TfR to CCPs. To examine this, we subjected ARPE-19 cells that stably express eGFP-clathrin to surface binding and uptake of A647-transferrin and rhodamine-EGF for 5 min and then imaging by TIRFM (Fig. 3*A*). We previously found this method to be robust for detecting changes in CCP cargo recruitment (15).

We examined these images through automated detection and analysis of clathrin-labeled structures (CLSs) using a Gaussian-based modeling approach (6), as was described in (15, 53, 54). We use the term CLS to denote clathrin structures detected in fixed samples, since identification of *bona fide* CCPs from short-lived sCLSs requires live-cell analysis (5, 6). This approach allows study of the effect of OGT silencing on the recruitment of specific cargo to CLSs, as well as sorting of CLSs by cargo type to determine if there may be cargo-specific effects of protein O-GlcNAc modification on clathrin structures.

Consistent with what we observed in experiments shown in Figures 1 and S1, OGT silencing resulted in a robust increase in eGFP-clathrin in individual CLSs (Fig. 3*B*), as well as an increase in A647-Tfn (Fig. 3*C*) and rhodamine-EGF (Fig. 3*D*) in these structures. As expected, eGFP-clathrin intensity levels within CLSs were significantly increased upon OGT silencing when considering multiple independent experiments (Fig. 3*E*), and this effect was observed in all cohorts of CLS when sorted by Tfn or EGF content (Fig. 3*F*). This suggests that the increase in CCP size observed upon OGT silencing may not be specific to CCPs harboring EGFR *versus* TfR.

The recruitment of specific receptors to CCPs upon OGT silencing may either scale with the size of CCPs, suggesting that OGT does not impact cargo recruitment separately from the control of CCP assembly or may alter the proportion of receptor recruitment to clathrin structures. To distinguish between these possibilities, we examined the levels of A647-Tfn or rhodamine-EGF in individual CLSs. Interestingly, the ratio of A647-Tfn to eGFP-clathrin was reduced in OGT-silenced cells, as seen in individual CLSs in a representative experiment (Fig. 3*G*, *left panel*), as well in multiple independent experiments (Fig. 3*G*, right panel). In contrast, the ratio of rhodamine-EGF to eGFP-clathrin was not impacted by OGT silencing (Fig. 3*H*). This suggests that OGT silencing results in larger CCPs that proportionally recruit more EGFR yet are less effective at proportional recruitment of TfR.

### O-GlcNAc regulation of specific endocytic accessory proteins

**Figure 4:**
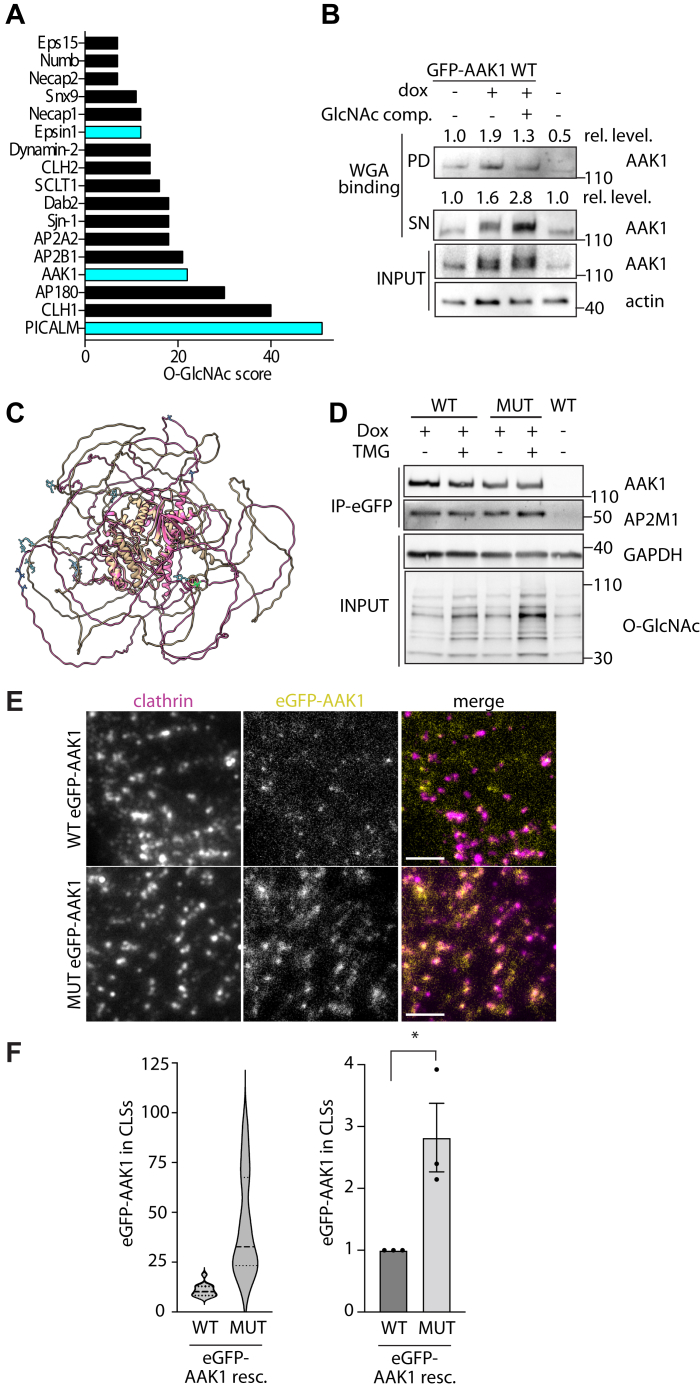
**Mutation of predicted O-GlcNAc modification sites alters CCP recruitment of AAK1**. *A*, shown is the result of the analysis of (57) showing the probability of O-GlcNAc modification of each endocytic protein (O-GlcNAc score). Highlighted in *teal* are the three proteins (Epsin1, AAK1, and CALM) examined in further detail here. *B*, following treatment with doxycycline (dox) to induce expression of eGFP-AAK1, samples were subjected to WGA pull-down and Western blotting as indicated. Some samples (designated ‘GlcNAc comp’) had WGA beads preincubated with an excess 1M GlcNAc to competitively block the binding of O-GlcNAc–modified proteins to WGA beads. Also indicated at the relative levels (‘rel. level”) of bands, quantified by densitometry measurements of blots as indicated. PD: pull-down fraction, SN: supernatant fraction. *C*, the structure of AAK1 predicted by AlphaFold, as per Experimental procedures. This structure was further decorated with O-GlcNAc at the predicted residues (shown in *teal*); however, the O-GlcNAc modification was not taken into consideration at the time of structure determination. *C*–*E*, ARPE-19 cells were engineered to stably harbor a transgene for inducible expression of eGFP-tagged AAK1 WT or harboring mutations in the eight predicted O-GlcNAc modification sites (mut). *D*, following treatment with doxycycline (dox) to induce expression of eGFP-AAK1, samples were subjected to immunoprecipitation of eGFP-AAK1 and Western blotting as indicated. *E* and *F*, cells were treated with siRNA to silence endogenous AAK1. Following siRNA transfection to silence endogenous AAK1 and treatment with dox, cells were fixed and stained to label clathrin heavy chain, followed by imaging by TIRF and epifluorescence microscopy. Scale bar represents 3 μm. *F*, images as in (*E*) were subjected to automated detection and analysis of CLSs as described in Experimental procedures. Shown are the measurements of eGFP-AAK1 intensity within CLSs, as the distribution of these values in a representative experiment as a violin plot (*left panel*), or the mean (bar) ± SE from three independent experiments, as well as individual values from each experiment (dots). The total number of CLSs and individual cells (respectively) quantified are as follows: eGFP-AAK1 WT 9645, 33; eGFP-AAK1 mut 9576, 42 (total) ∗*p* < 0.05. All Western blot panels show approximate molecular weight in kDa. CCP, clathrin-coated pit; O-GlcNAc, O-linked β-GlcNac; AAK1, AP2-associated kinase-1; TIRF, total internal reflection fluorescence; WGA, wheat germ agglutinin.

Direct O-GlcNAc modification of specific proteins or specific residues has been difficult to resolve (55, 56). Methods to predict the O-GlcNAc modification of specific proteins recruited to CCPs are particularly useful. A recent comprehensive study that predicted O-GlcNAc modification of specific proteins revealed that many clathrin endocytic accessory proteins show likelihood of O-GlcNAc modification (57) (Fig. 4*A*), although it is not clear if this represents overrepresentation of endocytic proteins within the cohort of predicted O-GlcNAc–modified proteins. Interestingly, proteins with significant intrinsically disordered regions such as AAK1, CALM/AP180, and Epsin are within this list, consistent with proteins with intrinsically disordered regions being preferred targets for O-GlcNAc modification (58). The possibility that O-GlcNAc modification regulates AAK1, CALM, and/or Epsin is consistent with central roles that these proteins play in the formation of CCPs and agrees with the effects we observed upon disruption of cellular O-GlcNAcylation on CCP formation (Figs. 1 and 2). Furthermore, AAK1 was previously shown to be modified by OGT in an OGT array, with four AAK1 serine/threonine sites modified with O-GlcNAc, although the specific residues were not identified (51). Hence, we focused our efforts on elucidating the possible regulatory capacity of O-GlcNAc modification of AAK1.

We sought to examine if AAK1 may be O-GlcNAc modified in cells. To support the detection of O-GlcNAc modification of AAK1, we developed a cell line that stably expresses eGFP-tagged AAK1 using the Sleeping Beauty transposon system (59), which allows modest overexpression of AAK1 (∼2× endogenous) (Fig. S3). In these cells, eGFP-AAK1 expression is induced upon treatment with doxycycline (dox). Upon induction of eGFP-AAK1 expression, we performed a wheat germ agglutinin (WGA) pull-down assay, allowing identification of proteins modified with O-GlcNAc and other glycans. As expected, AAK1 was detected in the WGA pull-down at a higher level in cells treated with doxycycline to induce eGFP-AAK1 expression (Fig. 4*B*). Importantly, preincubation of WGA beads with free GlcNAc decreased the AAK1 detected in the pull-down (GlcNAc comp., Fig. 4*B*), suggesting that the detection of AAK1 in the pull-down is specific for O-GlcNAc–modified AAK1. Consistent with this, preincubation of WGA beads with free GlcNAc retained substantially more AAK1 in the supernatant (Fig. 4*B*). This result is consistent with the previous determination of AAK1 O-GlcNAc modification (51) and suggests that a significant portion of AAK1 is subject to O-GlcNAc modification under these conditions.

Data from the mouse synaptosome revealed AAK1 (Q3UHJ0, AAK1_MOUSE) had seven O-GlcNAc sites based on a lectin chromatography peptide analysis (60) of which five aligned with human AAK1 (Q2M2I8, AAK1_HUMAN). We also identified three other O-GlcNAc sites from a new advanced O-GlcNAc database v1.1 (https://www.oglcnac.mcw.edu/) (57). These eight sites fall in the QP-rich region of AAK1 and not in the AP2 interaction domain. In total, we identified eight serine/threonine residues within human AAK1 that are likely to be modified with O-GlcNAc: S447, T507, S519, T359, T360, T448, T445, S650. Since experimentally determined structures of AAK1 are currently restricted to its kinase domain, we used AlphaFold2 (61, 62) to predict the structure of full-length AAK1 (Fig. 4*C*). The predicted AAK1 structure was consistent with the experimentally determined structures of the AAK1 kinase domain; alignment of the peptide backbone of the predicted kinases domains and the experimentally determined domains have RMSD values between 0.438 and 0.794 Å (Fig. S2). This full-length predicted structure revealed that surrounding the highly ordered core of AAK1 that includes the kinase domain, a large portion of the protein, is predicted to be comprised of intrinsically disordered regions. Importantly, the residues predicted to be O-GlcNAc modified are found within the intrinsically disordered regions of AAK1 (Fig. 4*C*, *O-GlcNAc modification in teal*).

To determine the contribution of specific putative O-GlcNAc–modified sites in AAK1 to its function and to CCP formation, we developed a knockdown-rescue approach using a mutant of AAK1 (fused to eGFP) in which all eight of these predicted Ser/Thr sites were mutated to Ala (Fig. S3). Mutating several potential O-GlcNAc sites in a clustered region is essential as it fits the progressive pattern of OGT modification of its substrates and its reaction mechanism (60). Using the sleeping beauty transposon system, we detect WT or mutant eGFP-AAK1 expression upon the rescue of AAK1 expression with doxycycline treatment (Fig. S4).

To assess how the mutations in putative O-GlcNAc modification sites may impact AAK1 function, we first examined its association with AP2, a well-established interactor of AAK1 (18). We immunoprecipitated eGFP-AAK1 WT or mutant and probed for the μ2-subunit of AP2 (Fig. 4*D*). We detected no differences in the extent of AP2 association with the AAK1 mutant compared to WT AAK1. This indicates that mutation of putative O-GlcNAcylation sites on AAK1 does not grossly disrupt its structure or association with AP2, consistent with a computational analysis of the effect of the mutations showing minimal impact on AAK1 secondary structure (Fig. S5).

To elucidate how the putative O-GlcNAc modification sites impact AAK1 recruitment to clathrin structures, we silenced endogenous AAK1, followed by induction of eGFP-AAK1 proteins, immunostaining to label clathrin, and imaging by TIRF and epifluorescence microscopy (Fig. 4*E*). We examined these images with automated detection and analysis of CLSs and found that mutant eGFP-AAK1 was recruited to clathrin structures much more robustly than WT eGFP-AAK1, both when examining the distribution of individual CLSs in a single representative experiment (Fig. 4*F*, *left panel*) and in multiple independent experiments (Fig. 4*F*, *right panel*). While there was no significant difference between eGFP-AAK1 WT and mutant-expressing cells with respect to clathrin intensity per CLS (Fig. S6*A*), we observed a robust increase in clathrin intensity upon the slight overexpression of WT eGFP-AAK1 achieved by this knockdown-rescue strategy (Fig. S6*B*). This is consistent with an increase in AAK1 expression and recruitment to CCPs leading to an increase in CCP size and suggests that there is a limit to the ability of AAK1 to increase CCP size.

Collectively, these results indicate that the level of AAK1 recruited to clathrin structures may control the assembly of proteins into CCPs and thus the size of these structures. Furthermore, our results show that the mutation of the eight putative O-GlcNAc modification residues robustly increases AAK1 recruitment to clathrin structures, suggesting that OGT and O-GlcNAcylation of AAK1 may suppress AAK1 recruitment to CCP, although these results cannot exclude the possibility of other posttranslational modifications regulating AAK1. We attempted to detect O-GlcNAc modification of AAK1 by antibody-based detection of O-GlcNAc residues but were unable to resolve O-GlcNAcylation of AAK1 by this method, perhaps due to this approach having some sequence specificity (63). Nonetheless, our WGA pull-down of the WT eGFP-AAK1 and previous determination of AAK1 O-GlcNAc modification (51) and the functional effects on AAK1 recruitment to clathrin structures upon mutation of putative O-GlcNAc–modified residues (Fig. 4) suggest that AAK1 may be subject to O-GlcNAc modification to suppress AAK1 recruitment to CCPs.

**Figure 5:**
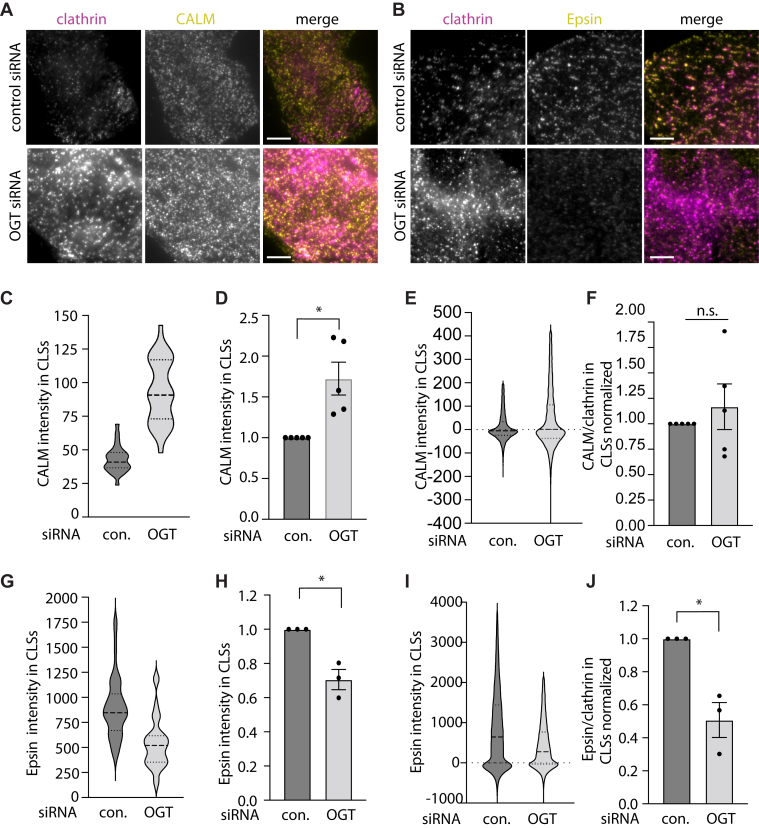
**OGT silencing distinctly impacts recruitment of CALM and Epsin to CCPs**. ARPE-19 cells stably expressing eGFP-clathrin light chain were treated with siRNA to silence OGT or nontargeting (control) siRNA, subjected to immunofluorescence staining to detect CALM or Epsin1, and then imaged using TIRF microscopy. Shown are representative TIRF images for CALM (*B*) or Epsin1 (*C*) experiments. Scale bar represents 5 μm. (*C*–*J*) Images as in (*A* and *B*) were subjected automated detection and analysis of CLSs as described in Experimental procedures. *C* and *D*, the measurements of CALM intensity within CLSs, shown as the distribution of these values in a representative experiment as a violin plot (*C*), or the mean (bar) ± SE from five independent experiments, as well as individual values from each experiment (dots) (*D*). *E* and *F*, the ratio of CALM/eGFP-clathrin intensities within CLSs, shown as the distribution of these values in a representative experiment as a violin plot (*E*), or the mean (bar) ± SE from five independent experiments, as well as individual values from each experiment (dots) (*F*). *G* and *H*, the measurements of Epsin1 intensity within CLSs, shown as the distribution of these values in a representative experiment as a violin plot (*G*), or the mean (bar) ± SE from five independent experiments, as well as individual values from each experiment (dots) (*H*). *I* and *J*, the ratio of Epsin1/eGFP-clathrin intensities within CLSs, shown as the distribution of these values in a representative experiment as a violin plot (*I*), or the mean (bar) ± SE from five independent experiments, as well as individual values from each experiment (dots) (*J*). The total number of CLSs and individual cells (respectively) quantified are as follows, for CALM staining: control siRNA 95762, 134; OGT siRNA 106993, 139; for Epsin1 staining: control siRNA 61718, 85; OGT siRNA 55792, 79 ∗*p* < 0.05. CCP, clathrin-coated pit; TIRF, total internal reflection fluorescence.

In addition to AAK1, CALM and Epsin were also identified as proteins with substantial composition of intrinsically disordered regions and a high likelihood of O-GlcNAc modification (Fig. 4*A*). We next examined how OGT silencing can impact the recruitment of CALM or Epsin to clathrin structures, using staining of each protein in cells expressing eGFP-clathrin, followed by TIRFM (Fig. 5, *A* and *B*) and CLS analysis. We previously validated Epsin antibodies (64) and validated CALM antibodies (Fig. S7) for use in TIRFM. The level of CALM recruited to CLSs was significantly increased upon OGT silencing when examining individual CLSs measurements in a representative experiment (Fig. 5*C*) and in multiple independent experiments (Fig. 5*D*). Since OGT silencing increases the recruitment of eGFP-clathrin in clathrin structures, we asked whether the increase in CALM recruitment to CLSs scales with clathrin. The ratio of CALM to eGFP-clathrin intensity in CLSs was unchanged upon OGT silencing when examining individual CLSs measurements in a representative experiment (Fig. 5*E*) and in multiple independent experiments (Fig. 5*F*). This suggests that while OGT silencing triggers an increase in CALM recruitment to CLSs, this increase in CALM may reflect an increase size of these structures and not a specific effect on CALM recruitment *per se*.

In contrast to the effect of OGT silencing on CALM recruitment, loss of OGT triggered a loss of Epsin from CLSs, when examining individual CLSs measurements in a representative experiment (Fig. 5*G*) and in multiple independent experiments (Fig. 5*H*). This loss of Epsin recruitment to CLSs was retained when examining the ratio of Epsin to clathrin (Fig. 5, *I* and *J*). This indicates that OGT does not act similarly on all endocytic accessory proteins; instead, while OGT and O-GlcNAcylation may suppress the recruitment of clathrin, AAK1, and CALM to clathrin structures, OGT and O-GlcNAcylation enhance Epsin recruitment to clathrin structures. The selective control of recruitment of different endocytic accessory proteins by OGT is consistent with cargo-selective effects on the recruitment of EGFR and TfR to clathrin structures (Fig. 3).

Our results indicate that loss of OGT and cellular O-GlcNAc protein modification suppresses eGFP-AAK1 recruitment to clathrin structures and leads to an increase in CCP size and loss of Epsin recruitment therein. To determine if AAK1 may be functionally required for the alterations in CCP size seen upon perturbation of OGT, we first examined the effect of inhibition of AAK1 by treatment with the small molecule inhibitor LP-935509. As expected, treatment with LP-935509 led to a loss of phosphorylation of the μ2 subunit of AP2 (Fig. S8), establishing effective inhibition of AAK1 kinase activity.

**Figure 6:**
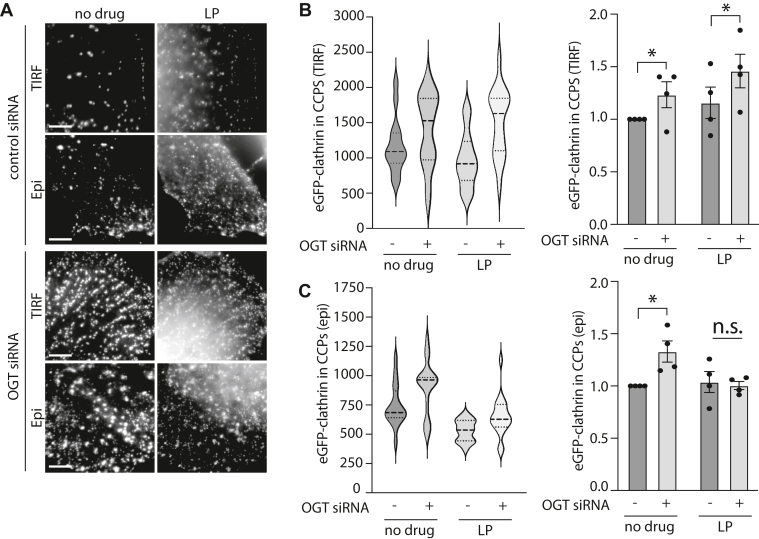
**Inhibition of AAK1 with LP-935509 reverses the effect of OGT silencing on CCP size.** ARPE-19 cells stably expressing eGFP-clathrin light chain were treated with siRNA to silence OGT or nontargeting (control) siRNA. Then, immediately prior to fixation, cells were treated with 5 μM LP-935509 (LP) or vehicle control (no drug) for 3 h and then imaged using TIRF and epifluorescence microscopy. *A,* shown are the representative images obtained by TIRF or epifluorescence microscopy. Scale bar represents 5 μm. Images were subjected to automated detection and analysis of CLSs as described in Experimental procedures. *B* and *C*, shown is the intensity of eGFP-clathrin within CLSs detected in TIRF (*B*) or epifluorescence (*C*) images, in each case showing the distribution of values within a single representative experiment as a violin plot (*left panels*) and the mean (bar) ± SE from five independent experiments, as well as individual values from each experiment (dots) (*right panels*). The total number of CLSs and individual cells quantified (respectively) are as follows: control siRNA, no drug 6335, 46; control siRNA, LP-935509, 9291, 76; OGT siRNA, no drug 9309, 70; OGT siRNA, LP-935509, 10,063, 76; ∗*p* < 0.05. AAK1, AP2-associated kinase-1; CCP, clathrin-coated pit; TIRF, total internal reflection fluorescence.

To determine if AAK1 contributes to the increase in CCP size upon loss of OGT, we examined the effect of LP-935509 treatment following OGT silencing on clathrin structures in cells expressing eGFP-clathrin by imaging with TIRF (Fig. 6*A*) and widefield epifluorescence (Fig. 6*A*) microscopy. Consistent with our results so far, automated detection and analysis of CLSs in TIRFM images showed that OGT silencing triggered an increase in eGFP-clathrin intensity in CLSs, and this was not affected in cells treated with LP-935509 (Fig. 6*B*). Since the intensity of eGFP-clathrin within CLSs in TIRF images can be impacted by both the amount of eGFP-clathrin molecules recruited per structure or the curvature of CLSs, we also examined the intensity of CLSs in widefield epifluorescence microscopy images, which are only impacted by the amount of eGFP-clathrin per CLS and not their curvature. While OGT silencing increased eGFP-clathrin intensity in CLSs in epifluorescence images, this effect was ablated by treatment with LP-935509 (Fig. 6*C*). The fact that the intensity of eGFP-clathrin in CLSs in TIRF images remains increased upon treatment with LP-935509, while this value is suppressed in widefield epifluorescence images, suggests that LP-935509 treatment impairs both the increase in size of CLSs upon OGT silencing and the acquisition of curvature of these structures. Taken together, these results indicate that AAK1 is required for the increase in clathrin structure size upon loss of OGT, suggesting that the increased recruitment of AAK1 to clathrin structures may be functionally required for the alterations in clathrin structure assembly seen upon loss of OGT.

### Nutrient availability impacts O-GlcNAc protein modification and clathrin structure formation

**Figure 7:**
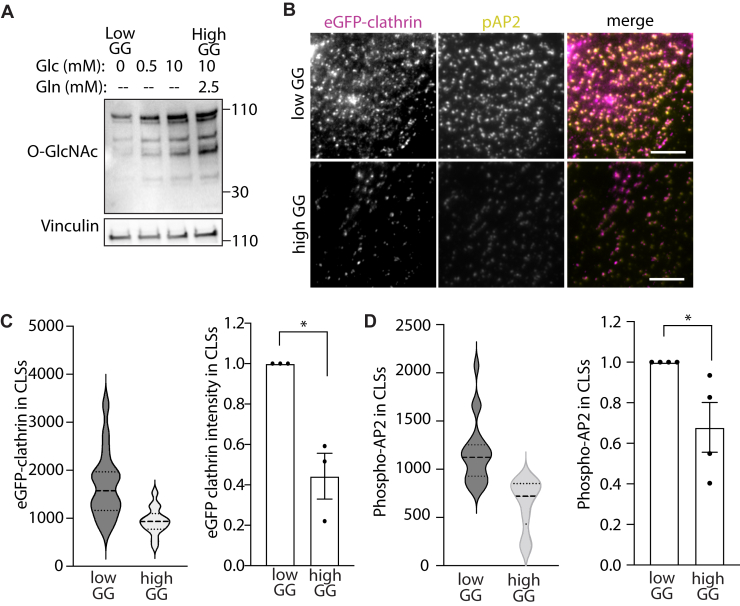
**Nutrient availability impacts O-GlcNAcylation and CCP size.** ARPE-19 cells stably expressing eGFP-clathrin were incubated in media containing glucose and glutamine as indicated for 4 h (low GG: 0 mM glucose and 0 mM glutamine; high GG: 10 mM glucose and 2.5 mM glutamine). *A*, immunoblots of whole cell lysates probed with antibodies as indicated. All Western blot panels show approximate molecular weight in kDa. *B*, following low GG/high GG treatment for 4 h, cells were fixed, subjected to staining to detect phosphorylated AP2, and then imaged by TIRF microscopy. Scale bar represents 5 μm. *C* and *D*, images as in (*B*) were subjected to automated detection and analysis of CLSs as described in Experimental procedures. Shown is the intensity of eGFP-clathrin (*C*) or pAP2 (*D*) within CLSs detected in TIRF microscopy images, in each case showing the distribution of values within a single representative experiment as a violin plot (*left panels*) and the mean (bar) ± SE from three independent experiments, as well as individual values from each experiment (dots) (*right panels*). The total number of CLSs and individual cells quantified (respectively) are as follows: low GG 45763, 69; high GG 45544, 66; ∗*p* < 0.05. CCP, clathrin-coated pit; O-GlcNAc, O-linked β-GlcNac; TIRF, total internal reflection fluorescence.

The impact of OGT and cellular protein O-GlcNAc modification suggest that signals that regulate O-GlcNAc modification may exert control over CCP formation. Cellular protein O-GlcNAcylation is highly sensitive to metabolic flux through the hexosamine biosynthetic pathway, and thus the availability of nutrients, in particular glucose and glutamine (65). We next aimed to resolve if the availability of glucose and glutamine may regulate CCP formation. To do so, we incubated cells for 4h in minimal media either devoid of glucose and glutamine (which we term “low GG”) or the same base minimal media supplemented with 10 mM glucose and 2.5 mM glutamine (which we term “high GG”). Comparing the levels of cellular O-GlcNAc modification shows that these conditions substantially impacted the total levels of cellular protein O-GlcNAc modification (Fig. 7*A*).

To determine how nutrient availability impacts CCP formation and AAK1 activity, we incubated cells stably expressing eGFP-clathrin for 4h in either low-GG or high-GG media. We then stained cells for phospho-AP2 (the substrate of AAK1), followed by imaging by TIRFM (Fig. 7*B*). We then examined images through automated detection and analysis of CLSs. Consistent with the effect of OGT silencing that increased CCP size, we observed that low-GG conditions that exhibit reduced protein O-GlcNAcylation had a significantly elevated intensity of eGFP-clathrin in CLSs, as observed in CLSs measurements in a single representative experiment (Fig. 7*C*, *left panel*) as well as in multiple independent experiments (Fig. 7*C*, *right panel*). We observed similar results for phospho-AP2 (Fig. 7*D*). Together, these results indicate that the availability of glucose and glutamine exert significant effects on CCP formation that are correlated with the regulation of CCP formation by protein O-GlcNAcylation.

## Discussion

Understanding how cells adapt their endocytic membrane traffic to their environment under different metabolic conditions is central to understanding the contribution of membrane traffic under distinct physiological contexts. We investigated how the nutrient-responsive O-GlcNAc modification modulates clathrin-dependent endocytosis. We identified that O-GlcNAc affects the endocytosis machinery of CME using multiple independent approaches, including siRNA silencing of OGT, pharmacological manipulation of OGA, and use of a mutant of AAK1 that is predicted to lose O-GlcNAc modification. To our knowledge, we provide the first direct functional evidence that CME spatiotemporal dynamics are modulated by an intrinsic metabolic signal: protein O-GlcNAcylation. This provides further evidence as to the versatility and adaptability of endocytic traffic *via* O-GlcNAc modification.

The posttranslational modification with O-GlcNAc has been difficult to detect and quantify on a per protein basis, as it is highly dynamic, present at substoichiometric amounts on many nucleocytoplasmic proteins and is labile due to its chemical structure (55, 56). The current methods to detect and quantify O-GlcNAc modifications on proteins come with many limitations and it is difficult to identify specific sites of O-GlcNAc modification (56). We show that a significant portion of AAK1 may be O-GlcNAc modified under the conditions we examined using a WGA pull-down approach (Fig. 4*B*). In addition, the mutation of putative O-GlcNAc–modified residues in AAK1 altered the recruitment of AAK1 to CCPs (Fig. 4, *E* and *F*). While these results suggest that one or more of the eight residues that we examined through mutagenesis may be O-GlcNAc modified, it is also possible that other posttranslational modifications of AAK1 occur on these residues. Recently, a similar mutagenesis strategy was used (66) for creating mutants of galectin 3 that altered four residues predicted by *in silico* analysis to be O-GlcNAc modified, without also experimentally identifying the residues that were O-GlcNAc modified. Thus, the functional outcome of mutations of candidate O-GlcNAc sites is compelling even in the absence of evidence of O-GlcNAc modification of specific residues (52).

AAK1 was previously shown to be subject to O-GlcNAc modification (51, 60). While the specific residues that are subject to O-GlcNAc modification are not known, we found that a mutant of AAK1 with eight predicted O-GlcNAc modification Ser/Thr residues changed to Ala exhibited significant alterations in recruitment to CCPs (Fig. 4). Specifically, the mutant AAK1 exhibited robustly increased recruitment to CCPs despite expression at similar levels as WT AAK1 (Fig. S4). Based on the findings in cells expressing eGFP-AAK1, loss of O-GlcNAc modification in the mutant AAK1 leads to enhanced recruitment of AAK1 to CCPs, which would be expected to enhance the phosphorylation of AP2 and promote CCP assembly and stabilization (5, 21). Hence, the loss of the putative O-GlcNAc modification sites on AAK1 leading to increased AAK1 localization to clathrin structures is consistent with the enhanced initiation rate and size of CCPs that we observed upon silencing OGT.

Notably, even the mild overexpression of the WT eGFP-AAK1 increased AP2 phosphorylation and CCP size, which was not further enhanced by expression of the eGFP-AAK1 with mutations in the putative O-GlcNAcylation sites (Fig. S6). Hence, these results suggest that increased recruitment of AAK1 to CCPs does increase AP2 phosphorylation and CCP size, but that there could be a limit to the extent of this increase that is already reached by the levels of AAK1 recruited to CCPs in the WT eGFP-AAK1–expressing cells. For example, this limit could reflect complete phosphorylation of AP2 complexes within CCPs when a certain level of AAK1 has been recruited therein. Taken together, these results suggest a model in which that loss of O-GlcNAc modification of AAK1, either by mutation of putative O-GlcNAcylation sites on AAK1 or by silencing OGT, may promote the recruitment of AAK1 to CCPs, which in turn may promote AP2 phosphorylation, enhance CCP assembly, and/or increase CCP size.

The putative O-GlcNAc modification sites are in regions of AAK1 that are predicted to be intrinsically disordered. The mutagenesis of eight Ser/Thr sites to Ala did not affect the predicted disordered regions of AAK1, indicating that the structure of AAK1 was not grossly impacted in this mutant, which is corroborated by the interaction of the mutant AAK1 with AP2 being retained (Fig. 4*D*). It is not clear how O-GlcNAc modification of AAK1 may regulate the recruitment of this kinase to CCPs. It may be tempting to speculate that the intrinsically disordered regions of AAK1 could contribute to liquid-liquid phase separation within CCPs and that O-GlcNAcylation of AAK1 may thus limit AAK1 partitioning within such a structure. It is also possible that O-GlcNAcylation of AAK1 may alter secondary structure or otherwise alter protein–protein interactions of AAK1 with clathrin-resident proteins other than AP2. Future research into the mechanism by which AAK1 O-GlcNAcylation impacts the function of this kinase in the regulation of CME will be very informative.

While our results suggest that O-GlcNAc modification of AAK1 may regulate the recruitment of this kinase to CCPs and thus the properties of CCPs, it is also possible that other CCP-resident proteins are modified with O-GlcNAc, impacting CCP properties and CME. It is also possible that O-GlcNAc–based regulation of CCP dynamics may be indirect, with broad alterations in proteins distal to CCPs impacting endocytic assemblies by other mechanisms such as changes in gene expression. While a systematic analysis of all CCP proteins to resolve other possible O-GlcNAc modifications is beyond the scope of this study, future research that can further resolve how O-GlcNAc posttranslational modification may regulate CME and endocytic membrane traffic will also reveal key insights into the metabolic control of membrane traffic.

Our results suggest that CCP initiation, assembly, and stabilization, as well as cargo recruitment, is robustly controlled by protein O-GlcNAcylation. Interestingly, another study found that the clathrin-dependent internalization was unaffected by silencing of OGT or OGA (66). In contrast, we found that suppression of OGT and OGA substantially impacted CCP initiation and assembly. Specifically, suppression of OGT enhanced CCP initiation and size, which would be expected to contribute to enhance endocytosis. At the same time, OGT silencing also impacted CCPs in a manner expected to suppress endocytosis, as seen by decreased CCP lifetimes that suggest enhanced abortive turnover of CCPs without internalization and reduced TfR recruitment to CCPs. This suggests that the effect of regulation of CCPs by O-GlcNAc modification can have complex effects that warrant further study.

We found that while OGT silencing increased the rate of CCP initiation, treatment of cells with TMG that leads to enhanced O-GlcNAc protein modification did not significantly change this parameter. This lack of effect of TMG treatment on CCP initiation may reflect the significant portion of AAK1 that appears to already be modified with O-GlcNAc in the conditions examined prior to TMG treatment (Fig. 4*B*). As such, the further enhancement of O-GlcNAc modification of AAK1 and other proteins may have limited effects on CCP initiation but impact subsequent assembly, stabilization, or turnover of CCPs. Nonetheless, the effects of OGT silencing that reduce protein O-GlcNAc modification and TMG treatment that enhance protein O-GlcNAc modification are largely complementary and suggest regulation by O-GlcNAc modification of several aspects of CCP assembly, stabilization, and cargo recruitment.

These changes in CCP initiation, assembly, and lifetime may reflect that OGT and OGA perturbations impact CCP dynamics in a manner to limit changes to the internalization of some receptors under a range of metabolic conditions. Alternatively, or in addition, the integration of effects controlled by O-GlcNAc modification may have limited effects on the internalization of some cargo (*e.g.* TfR), while significantly impacting the internalization of others. Consistent with this, inhibition of AAK1 leads to modest (∼25%) inhibition of TfR internalization (5, 21), while AAK1 may have much more significant control over that of LRP6 (67).

Our observation that the recruitment of EGFR to CCPs upon OGT silencing scales with the amount of clathrin, while that of TfR does not, suggests that O-GlcNAc modification may selectively impact the internalization of EGFR and TfR. This possible difference in regulation of CME of TfR and EGFR by protein O-GlcNAcylation could occur as a result of direct and distinct O-GlcNAc of modification of each receptor, as has been reported for EGFR (68). Alternatively, as TfR and EGFR reside largely in distinct CCPs (15), EGFR and TfR internalization may be differently regulated by O-GlcNAc modification as a result of distinct outcomes on subsets of CCPs. Interestingly, O-GlcNAc modification of hepatocyte growth factor–regulated tyrosine kinase substrate (HGS) alters EGFR intracellular traffic by suppressing ligand-stimulated EGFR degradation, leading to sustained signaling (69). As CCPs at the plasma membrane are also important regulators of EGFR signaling (15, 53, 54, 64, 70, 71), protein modification by O-GlcNAc may act to regulate EGFR membrane traffic and signaling at multiple levels.

Resolving how the robust modulation of CCP initiation, size, and lifetimes by O-GlcNAcylation impacts the internalization and membrane traffic of the diverse proteins at the cell surface will require systematic methods to study cell surface protein abundance. Such approaches have been done to study the impact of AP2 perturbation (72) or AMPK activation (39). Using similar approaches to study the effect of OGT or OGA perturbation may allow identification of a cohort of cell surface proteins that exhibit internalization with preferential regulation by protein O-GlcNAcylation.

In conclusion, we find that protein O-GlcNAc modification exerts control over the formation, assembly, and maturation of CCPs. As such, O-GlcNAc modification may be an important mediator of the control of endocytic membrane traffic by cellular metabolic state (13, 14). Endocytic membrane traffic controls the access of myriad cell surface proteins to their substrates in the extracellular milieu. Thus, further resolving how metabolic signals that impact protein O-GlcNAc modification and other mechanisms control cell surface membrane traffic will continue to be an important area of investigation.

## Experimental procedures

### Materials

Details about antibodies used for Western blotting and immunofluorescence experiments are in Table S1. Fluorophore-conjugated or horseradish peroxidase secondary antibodies were from Jackson ImmunoResearch. Rhodamine-EGF was generated in-house as previously described by (73).

### Cell culture

WT human retinal pigment epithelial (ARPE-19) cells were cultured in Dulbecco’s Modified Eagle’s Medium/F-12 (DMEM/F12; Thermo Fisher Scientific) supplemented with 10% fetal bovine serum (Thermo Fisher Scientific) and an antibiotic cocktail consisting of 100 U/ml penicillin and 100 μg/mg streptomycin (Thermo Fisher Scientific). Cells were incubated at 37 °C and 5% CO_2_ and 95% O_2_ until they reached 80% confluency and were suitable for passaging. Cells were passaged every 2 to 3 days, washed with sterile PBS (Sigma Aldrich), and lifted with 0.25% Trypsin-EDTA (Thermo Fisher Scientific).

### Stable transfections using sleeping beauty transposon system

pSBtet-BP was a gift from Eric Kowarz (Addgene plasmid # 60496; http://n2t.net/addgene:60496; RRID:Addgene_60496) (59). pCMV(CAT)T7-SB100 was a gift from Zsuzsanna Izsvak (Addgene plasmid # 34879; http://n2t.net/addgene:34879; RRID:Addgene_34879) (74). An oligonucleotide-encoding eGFP fused to AAK1 were generated by BioBasic Inc, using the sequence of eGFP, followed by the sequence encoding a spacer peptide (GGG GGG TCT GGT GGC AGT GGA GGG GGA TCC), followed by the sequence of human AAK1, as per accession number NM_Q2M2I8. This oligonucleotide sequence was subcloned into pSB-tet-BP to generate pSB-tet-BP-eGFP-AAK1(WT). From this plasmid, mutant AAK1 (S447, T507, S519, T359, T360, Thr448, Thr445, Ser650 to Ala) constructs were derived by site-directed mutagenesis by BioBasic Inc.

pSBtet-BP plasmids encoding various AAK1 WT and AAK1 mutant constructs alongside pCMV(CAT)T7-SB100 were cotransfected into ARPE-19 cells using FuGene HD transfection reagent, as per manufacturer’s protocol (Promega), followed by a selection of stably engineered cells in media supplemented with 2 μg/ml puromycin for a period of 2 to 3 weeks.

pSBtet-BP stable cells were kept in cell culture media as indicated above but were incubated with 10% fetal bovine serum without tetracycline and maintained in 2 μg/ml puromycin. For the induction of AAK1-GFP WT or mutant, doxycycline (Dox) was used at a final concentration of 1 μM in the cell culture media for 24 h before freezing cells for different downstream applications.

### Western blotting

After incubation and pharmacological treatments as indicated, cells were rapidly cooled by washing with ice-cold PBS and then lysed in 2X-Laemmlli Sample buffer (0.5 M Tris pH 6.8, glycerol, 10% SDS) supplemented with 1 mM sodium orthovanadate, 10 mM okadaic acid, and 20 mM of protease inhibitor. Whole-cell lysates were then syringed 5 times with a 27.5-gauge syringe. Lysates were then heated at 65 °C for 15 min under reducing conditions (supplementation of lysis buffer with 10% beta-mercaptoethanol and 5% bromophenol blue), resolved by SDS-PAGE, and transferred to 0.2 m pore PVDF membrane (PALL Life Science). The membrane was blocked with 3% bovine serum albumin (BSA) and then incubated with indicated antibodies in 1% BSA at 1:1000 dilution at 4 °C overnight. The membrane was then subjected to a secondary horseradish peroxidase–conjugated antibody (with 1% BSA) and left to shake for 1 h at RT before imaging. Bands were visualized with Luminata ECL chemiluminescence substrate (Milipore Sigma).

### siRNA transfections

ARPE-19 cells were subject to siRNA gene silencing for a target (OGT, CALM, AAK1) and nontargeting control. Custom siRNAs were synthesized to target specific transcripts with sense strand sequences as follows: OGT-1 (referred to simply as “OGT” unless otherwise specified): GAA GAA AGU UCG UGG CAA A (UU), OGT-2: CCU GAU AGA UCU GGC AAU A (UU), CALM: CCU CAU ACC UCU UUA ACA A (UU), AAK1: GGG AAA GUC AGG UGG CAA U (UU). siRNA gene silencing was performed using Lipofectamine RNAiMAX (Life Technologies), as per the manufacturer’s instructions and as previously described (53). Each siRNA construct was transfected at 220 pmol/l precomplexed and incubated in the transfection reagent in Opti-MEM (Gibco) for 4-h. After the 4-h incubation, cells were washed and replaced in a regular 10% FBS DMEM/F12 growth medium. siRNA transfections were performed twice (72 h and 48 h) except for OGT, which was performed once, before whole-cell lysate preparation.

### Purification of GFP-fusion proteins

*Escherichia coli* BL21 cells transformed with GFP-binding V_H_H domain, GFP-binding protein (GBP) were in pHEN6 vector were a gift from Dr Greg Fairn (Dalhousie University). The cells were induced with 1 mM IPTG for 3 to 4 h in 30 °C. Bacterial cells were harvested by centrifugation (10 min at 3000g) and the pellet was resuspended in lysis buffer (50 mM Tris pH 7.5, 300 mM NaCl, 10 mM imidazole, 5% (v/v) glycerol). The cell suspension was sonicated at 4 °C for 2 min with a total of 20 s on and 59.9 s off with 15% amplitude. After centrifugation (20 min at 14,000 rpm), the soluble protein was loaded onto preequilibrated 1 ml NiNTA resin and purified. The His-tagged GBP was eluted by using NiNTA elution buffer (20 mM Tris pH 7.5, 300 mM NaCl, 250 mM imidazole, 5% (v/v) glycerol). Elution fractions containing GBP were pooled and dialyzed with Slide-A-Lyzer Dialysis Cassette G2 (3500 MWCO, 3 ml capacity) into Dulbecco’s Phosphate Buffered Saline (D8537) to remove the imidazole. The protein concentration was measured with a BCA.

The purified GBP was coupled to N-hydroxysuccinimide–activated Sepharose 4 Fast Flow (GE Healthcare) as described previously by (75) with certain modifications.

The coupling solution/medium were incubated at 0.5:1 while rotating for 3 h at room temperature. After coupling, the nonreacted groups on the medium were blocked by 0.1 M Tris–HCL, pH 8.5 for 2 to 3 h. The washes were alternatively between two different buffers (high and low pH) 0.1 M tris-buffered saline pH 8.0 and 0.1 M acetate 500 mM NaCl pH 4.5. The final affinity medium, referred to as GFP-NanoTrap, was stored in TBS with 20% ethanol.

AAK1-GFP WT or AAK1-GFP mutant or eGFP-clathrin cells were seeded onto 10 cm dishes and once 80 to 85% confluent, they were homogenized in 400 μl cold lysis buffer (20 mM Tris/HCl, pH 7.5, 150 mM NaCl, 0.5 mM EDTA, 2 mM PMSF, 0.5% Nonidet P-40). After a centrifugation step (10 min at 14,000 rpm at 4 °C), the supernatant was adjusted with dilution buffer (20 mM Tris/HCl, pH 7.5, 150 mM NaCl, 0.5 mM EDTA, 2 mM PMSF) and incubated in 100 μl of GFP-NanoTrap. The GFP-NanoTrap and protein lysate was incubated overnight at 4 °C while rotating. The next day, the samples were washed with dilution buffer and then with wash buffer 2 (20 mM Tris/HCl, pH 7.5, 300 mM NaCl, 0.5 mM EDTA, 2 mM PMSF) right before elution. The elution of GFP-protein was done by adding laemmli sample buffer directly to the cell lysate and incubating it at 65 °C for 5 min. After this, the samples were resolved by Western blotting.

### WGA pull-down assay

pSBTet-AAK1-GFP WT ARPE-19 cells or WT ARPE-19 cells were seeded onto 10 cm dishes until 80 to 85% confluent. Then, cells were homogenized in 400 μl cold lysis buffer (20 mM Tris/HCl, pH 7.5, 150 mM NaCl, 0.5 mM EDTA, 2 mM PMSF, 0.5% Nonidet P-40, 25 mM TMG). After a centrifugation step (10 min at 14,000 rpm at 4 °C), the supernatant was incubated in 150 μl of WGA Agarose from Vector Biolabs (catalog #: VECTAL10235) and INPUT samples were collected. The agarose-bound WGA was prewashed with PBS containing 0.2% Nonidet P-40 (NP-40). The WGA and protein lysate were incubated overnight at 4 °C under rotation. Then, supernatant fractions were collected, followed by washing of agarose beads with 0.2% NP-40 in PBS. The O-GlcNAc–modified proteins were eluted from the WGA agarose using 1 M GlcNAc (Sigma-Aldrich) in 0.2% NP-40 in PBS at room temperature in Figure 4. After this, the samples were resolved by Western blotting, as described above.

### Experiment seeding and pharmacological treatments

ARPE-19 cells stably expressing eGFP-CLC were seeded onto glass coverslips the day before the experiment, and in some cases gone through siRNA transfection (Figure 1, Figure 2, Figure 3, Figure 5, Figure 6, Figure 7). pSBTet-AAK1-GFP WT and pSBTet-AAK1-GFP mutant cells were seeded onto glass coverslips the day before the experiment for siRNA of AAK1 in Figure 3. After two rounds of siRNA transfection to AAK1, doxycycline was added to the cells during the rest day. Cells were incubated for 4h at 20 μM TMG for or in vehicle (dH_2_0) prior to live-cell microscopy (Fig. 2). Cells were either incubated for 3h at 5 μM of LP-935509 or in vehicle control (0.1% (v/v) DMSO) prior to the fixation (Fig. 6). Following 1-h serum starvation, cells were treated with 20 ng/ml rhodamine EGF and 10 μg/ml A657-Tfn in combination (Fig. 3), followed by immediate fixation in 4% paraformaldehyde. For Figure 7, cells were seeded onto 6-well plates for 24 prior to incubation in media without glucose, glutamine, and no phenol red (catalog no. A1443001) supplemented with 10% dialyzed fetal bovine serum (Thermo Fisher Scientific), and glucose and/or glutamine, as indicated, for 4 h.

### Immunofluorescence staining

For detection of total cellular protein (all immunofluorescence experiments), after treatments, as indicated, cells were fixed with 4% paraformaldehyde for 30 min, followed by quenching of fixative in 100 mM glycine, cell permeabilization in 0.1% Triton X-100 (all solutions made in PBS), and then blocking in 3% BSA (Thermo Fisher Scientific). Subsequently, cells were stained with primary and secondary antibodies as indicated and retained within an aqueous medium for imaging by TIRFM.

### Microscopy and image analysis

#### Image acquisition

Microscopy was performed using a Quorum Diskovery microscope that is comprised of a Leica DMi8 microscope equipped with a 63×/1.49 NA TIRF objective with a 1.8× camera relay (total magnification 108×). Imaging was done using 488-, 561, and 637-nm laser illumination and 527/30, 630/75, and 700/75 emission filters. The microscope was operated in TIRFM or widefield epifluorescence microscopy modes, as indicated. Images were acquired using a Zyla 4.2Plus sCMOS camera (Hamamatsu). Fixed-cell TIRFM imaging was done at room temperature with samples mounted in PBS. For live-cell imaging experiments (Figs. 1 and 2), cells were maintained at constant 37 °C during imaging, in phenol-free DMEM/F12 media (Gibco) supplemented with 20 mM Hepes.

#### Detection and analysis of CCP dynamics in time-lapse image series

Automated detection, tracking, and analysis of CCPs (as in Figs. 1 and 2) was as previously described (4, 5, 6, 64) following time-lapse TIRFM of RPE cells stably expressing eGFP-CLCa. Diffraction-limited clathrin structures were detected using a Gaussian-based model method to approximate the point-spread function (6), and trajectories were determined from clathrin structure detections using u-track (76). sCLSs were distinguished from bona fide CCPs based on unbiased analysis of clathrin intensities in the early CCP stages (5, 6). Both sCLSs and CCPs represent nucleation events, but only *bona fide* CCPs represent structures that undergo stabilization, maturation, and in some cases scission to produce vesicles (5, 6). We report the sCLS nucleation, CCP initiation, CCP lifetime distribution, and the density of persistent CCPs, as well as the intensity of eGFP-CLC within structures as the ‘plateau intensity’ of eGFP-clathrin within these structures. CCPs exhibit several phases including initiation, growth/assembly, plateau, and disassembly/scission (77). Here, we define the ‘plateau intensity’ of eGFP-CLC in TIRF or epifluorescence microscopy images as the mean fluorescence of that protein within each detected clathrin structure, measured within timepoints corresponding to 30% and 70% of the total lifetime of that structure, during which time CCPs exhibit minimal growth or disassembly (64). Because CCPs are diffraction-limited objects, the amplitude of the Gaussian model of the fluorescence intensity of eGFP-CLC informs about CCP size (Figs. 1, *E* and *F* and 2, *E* and *F*). All measurements were subjected to ANOVA followed by Tukey post-test with a threshold of *p* < 0.05 for statistically significant differences between conditions.

#### Detection and analysis of CLS in fixed cell samples

CLSs were detected and quantified using custom software developed in Matlab (MathWorks), as previously described (Aguet *et al*., 2013; Garay *et al*., 2015) (64). Briefly, diffraction-limited clathrin structures were detected using a Gaussian-based model method to approximate the point-spread function of eGFP-CLCa or antibody-labeled clathrin in TIRF-M images. The fluorescence intensity corresponding to the secondary channel such as that of a second label (*e.g.* CALM, EPSIN, AAK1) or of clathrin within epifluorescence images within CLSs was determined by the amplitude of the Gaussian model for the appropriate fluorescent channel for each structure. As such, the measurements of protein intensity within CLSs represent enrichment of the corresponding signal relative to the local background fluorescence in the vicinity of the detected CLS. Measurements (mean levels of various proteins within specified CLS subset for each cell) were subjected to either two-sided student’s *t* test or ANOVA followed by Tukey post-test, with a threshold of *p* < 0.05 for statistically significant differences between conditions.

### Structure analysis of AAK1

AlphaFold 2 (61, 62) was used *via* collabfold (78) to predict the three-dimensional structure of AAK1 (961 amino acids) using the sequence NM_Q2M218 and an eight alanine (S447, T507, S519, T359, T360, T448, T445, S650) mutant sequence (mutant AAK1). Structures were visualized using ChimeraX (79, 80), and O-GlcNac residues were added by overlaying sugar onto S/T hydroxyl groups. Structural predictions of the low complexity regions were independently identified using Protein Disorder Prediction System Server (PrDOS) (81). A scatter plot was generated using the disorder predictions of both the WT and the mutant forms of AAK1. Experimentally determined crystal structures available to date of the kinase domain from human AAK1 were modeled to the predicted structure of AAK1 using ChimeraX (79, 80).

## Data availability

All data is contained within the article.

## Supporting information

This article contains supporting information (20, 82).

## Conflict of interest

The authors declare that they have no conflicts of interest with the contents of this article.
