# Supplementary material for: O-GlcNAc transferase modulates the cellular endocytosis machinery by controlling the formation of clathrin-coated pits
Source: J Biol Chem. 2023 Jan 31;299(3):102963. doi: 10.1016/j.jbc.2023.102963 (PMC9999237; doi:10.1016/j.jbc.2023.102963)
Supplement: Supporting information [file mmc1.pdf]

Supporting Information

O-GlcNAc transferase modulates the cellular endocytosis machinery by controlling the formation of clathrin coated pits

Sadia Rahmani [1,2], Hafsa Ahmed [1], Osemudiamen Ibazebo [1], Eden Fussner-Dupas [1,2], Warren W. Wakarchuk [3 #], Costin N. Antonescu[1,2 #]

1. Department of Chemistry and Biology, Toronto Metropolitan University, Toronto, ON M5B2K3
2. Graduate Program in Molecular Science, Toronto Metropolitan University, Toronto, ON M5B2K3
3. Department of Biological Sciences, University of Alberta, Edmonton, AB, T6G2E9

# Corresponding authors: Warren W. Wakarchuk, wakarchu@ualberta.ca, Costin N. Antonescu, cantonescu@torontomu.ca

Supplementary Information Contents:

Table S1: Antibodies and Chemicals

Figure S1: OGT silencing regulates the size of clathrin-coated pits
Figure S2: Overlay of AlphaFold generated AAK1 structures with experimentally resolved structures of AAK1
Figure S3: AAK1 residues predicted to be modified by O-GlcNAc and mutagenesis to generate O-GlcNAc deficient AAK1 mutant
Figure S4: Knockdown and rescue of AAK1
Figure S5: AAK1 WT and mutant display similar structure disorder prediction.
Figure S6: Slight overexpression of wild-type and GlcNAc deficient mutant of AAK1 similarly increase eGFP-clathrin intensity within clathrin labelled structures
Figure S7: Validation of CALM antibodies
Figure S8: LP-935509 treatment inhibits AP2 phosphorylation

Table S1: Antibodies and Chemicals detailed information.

Name	Company	Product code
**Antibodies**		
OGT/O-Linked-N-Acetylglucosamine Transferase	Abcam	ab177941
O-GlcNAc RL2	Abcam	
Anti-Phosphatidylinositol-binding clathrin assembly protein (Anti-PICALM)	Sigma Aldrich	HPA019061
AAK1	Cell Signaling Technology	79832
Phospho-AP2M1 (Thr156)	Cell Signaling Technology	#3843
Epsin	Abcam	ab75879
AP50 (AP2M1)	ThermoFisher Scientific	27355-1-AP
clathrin heavy chain antibody [X22]	Abcam	Ab2731
GAPDH	Cell Signaling Technology	14C10 #2118
Pan-Actin	Cell Signaling Technology	D18C11 #8456
**Chemicals**		
Thiamet G	Sigma Aldrich	SML0244 (Batch 22957)
LP-935509 (LP)	Axon MedChem	CAS [1454555-29-3] MF C20H24N6O3
D-(+)-Glucose solution 45% in H2O, sterile-filtered, BioXtra, suitable for cell culture	Sigma Aldrich	G8769
L-Glutamine BioXtra, suitable for cell culture	Sigma Alrich	G7513

DMEM, no glucose, no glutamine, no phenol red	ThermoFisher Scientific	A1443001

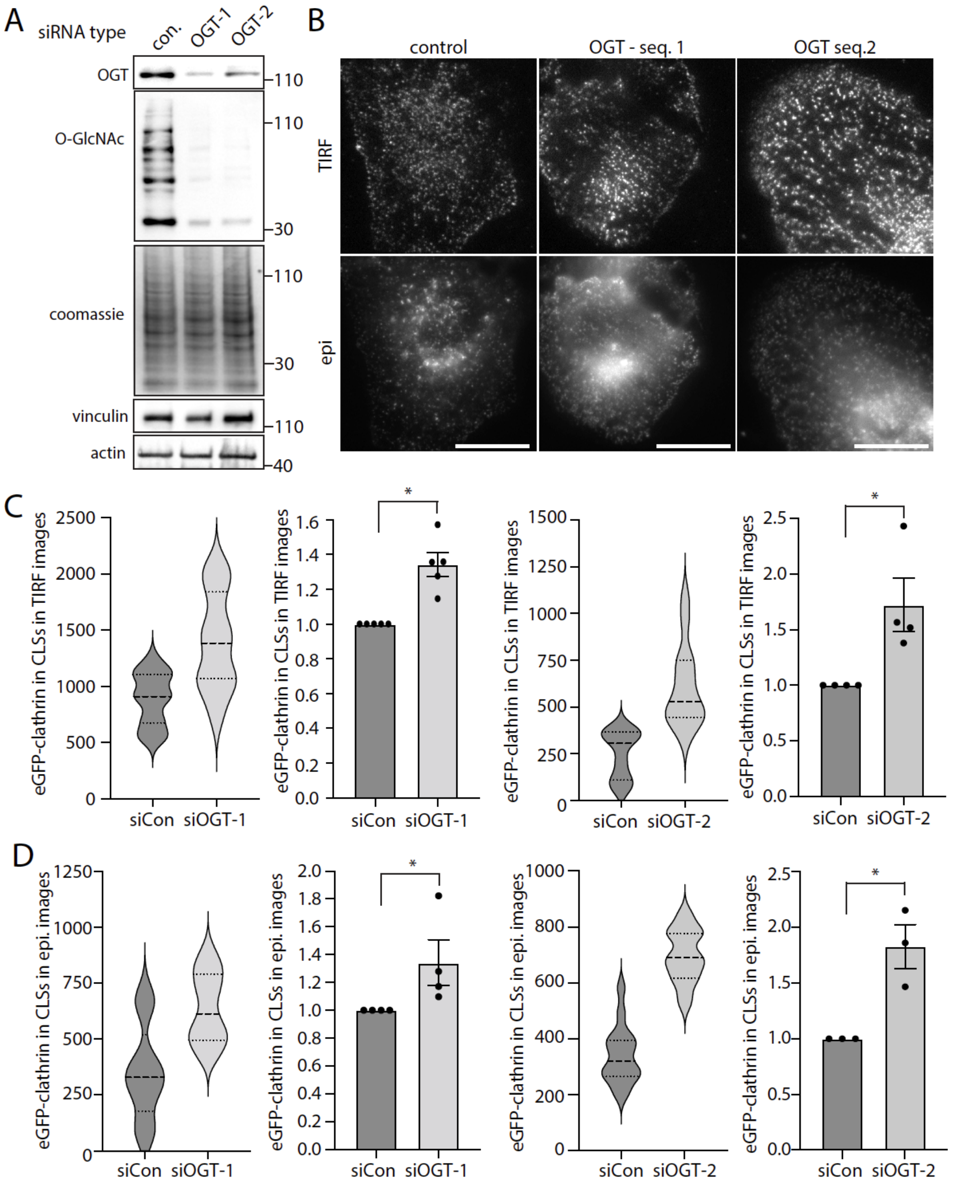

Figure S1. OGT silencing regulates the size of clathrin-coated pits. ARPE-19 cells stably expressing eGFP-clathrin light chain were treated with siRNA to silence OGT using two distinct oligos (OGT sequence 1, referred to simply as "OGT" throughout manuscript) or OGT sequence 2, or non-targeting (control) siRNA. (A) Shown are representative immunoblots of whole cell lysates probed with antibodies or subjected to Coomassie staining, as indicated. All western blot panels show approximate molecular weight in kDa. (B) Following siRNA transfection, cells were subjected fixation and then imaging using TIRF microscopy. Shown are representative TIRF and widefield epifluorescence microscopy images. Scale 5 µm. (C-D) Images as in (B) were subjected automated detection and analysis of CLSs as described in *Materials and Methods*. The measurements of eGFP-clathrin intensity within TIRF (C) or widefield epifluorescence (D) microscopy images is shown as the distribution of these values in a representative experiment as a violin plot, or the mean (bar) ± SE from 3-4 independent experiments, as well as individual values from each experiment (dots). The total number of CLSs quantified and cells analysed in 3-5 independent experiments are as follows: in (C) left panels for OGT sequence 1 silencing: control siRNA 8727, 74, OGT siRNA sequence 1: 1134, 75; in (C) right panels for OGT sequence 2 silencing: control siRNA 3827, 54, OGT siRNA sequence 2: 3457, 57; in (D) left panels for OGT sequence 1 silencing: control siRNA 8009, 58, OGT siRNA sequence 1: 7884, 61; in (D) right panels for OGT sequence 2 silencing: control siRNA 1493, 40, OGT siRNA sequence 2: 2995, 42. *, $p < 0.05$.

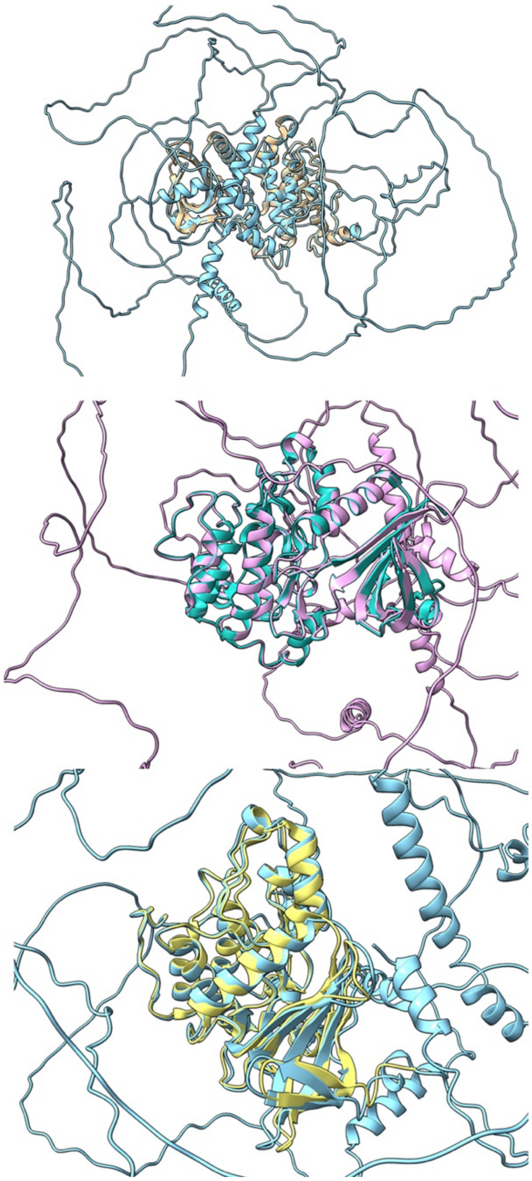

Figure S2. Overlay of AlphaFold generated AAK1 structures with experimentally resolved structures of AAK1. AAK1 wild type structure resolved by AlphaFold is compared to the experimentally resolved AAK1 structures: 4WSQ (PDB DOI: 10.2210/pdb4WSQ/pdb) (top panel), 5TE0 (PDB DOI: 10.2210/pdb5TE0/pdb) (middle panel), and 5L4Q (PDB DOI: 10.2210/pdb5L4Q/pdb) (bottom panel). The root mean squared deviation (RMSD) values for each 4WSQ, 5TE0, and 5L4Q as follows 0.557 Angstroms, 0.439 Angstroms, and 0.794 Angstroms respectively.

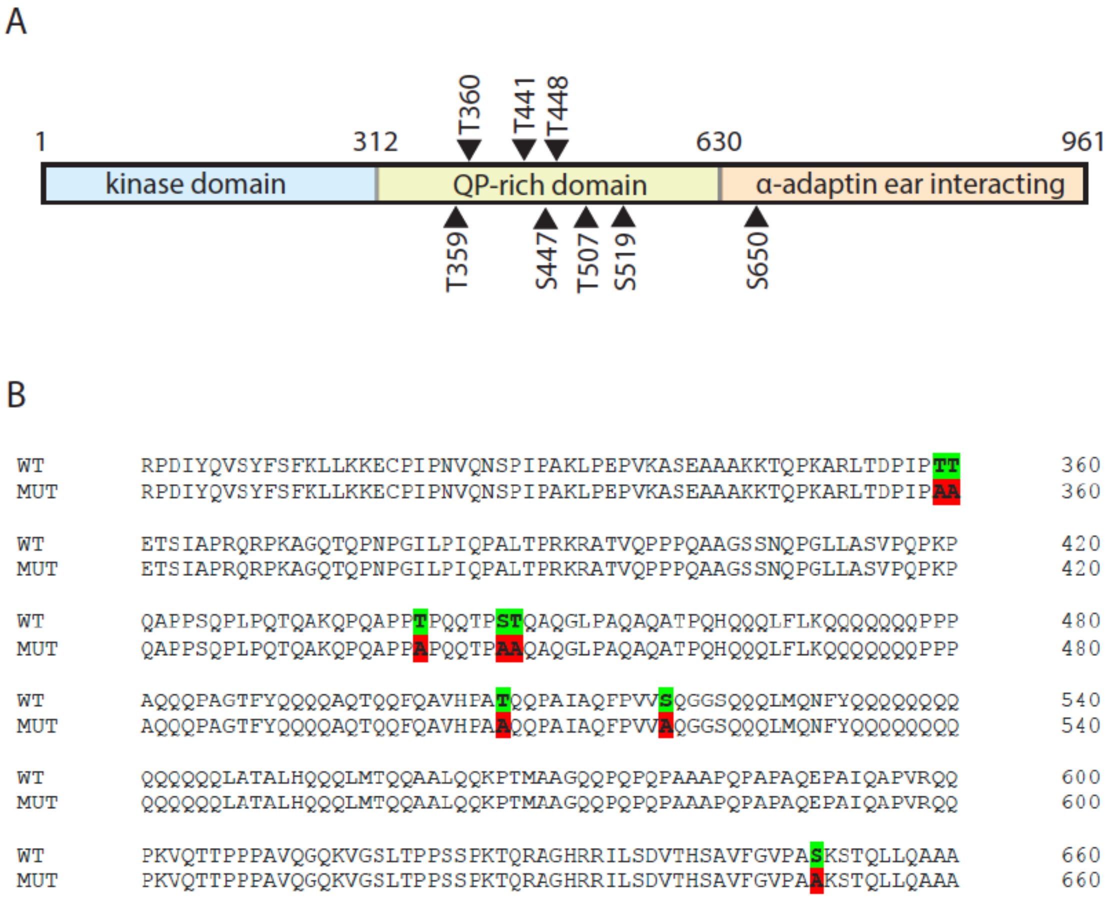

Figure S3. AAK1 residues predicted to be modified by O-GlcNAc and mutagenesis to generate O-GlcNAc deficient AAK1 mutant. (A) The domain structure of AAK1, a 961 amino acid protein that has three main domains: Kinase domain, QP rich domain and AP2 interaction domain. The predicted O-GlcNAc sites fall mainly in the QP rich domain. (B) We identified O-GlcNAc sites on AAK1, and mutated them to Ala: T359A, T360A, T441A, S447A, T448A, T507A, S519A, S650A.

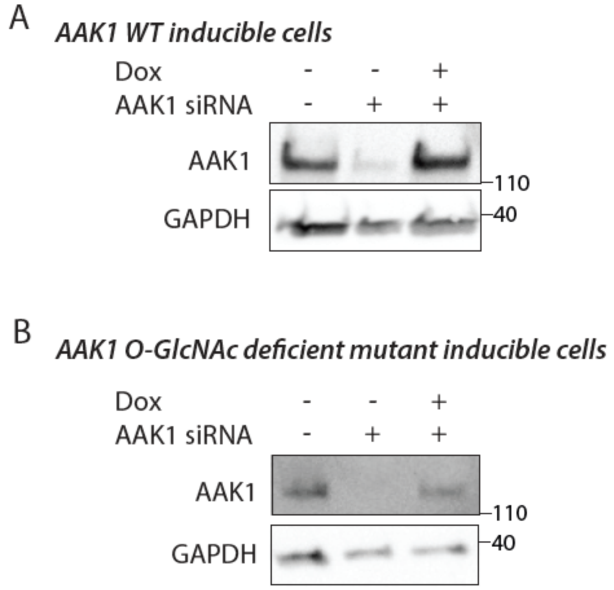

Figure S4. Knockdown and rescue of AAK1. ARPE-19 cells were engineered to stably harbor a transgene for inducible expression of eGFP-tagged AAK1 wild-type (WT) or harboring mutations in the 8 predicted O-GlcNAc modification sites (mut). Cells were treated with siRNA to silence endogenous AAK1 as shown. Following siRNA transfection and treatment with dox for 24h, whole cell lysates were resolved by western blotting. Shown are representative western blots using antibodies to detect AAK1 expression or GAPDH (loading control). This strategy achieves a slight overexpression of wild-type AAK1 and a near endogenous level of expression of mutant AAK1. The migration of the eGFP-tagged AAK1 (~165kDa) is similar to that of endogenous AAK1 (~145kDa) under these western blotting conditions. All western blot panels show approximate molecular weight in kDa.

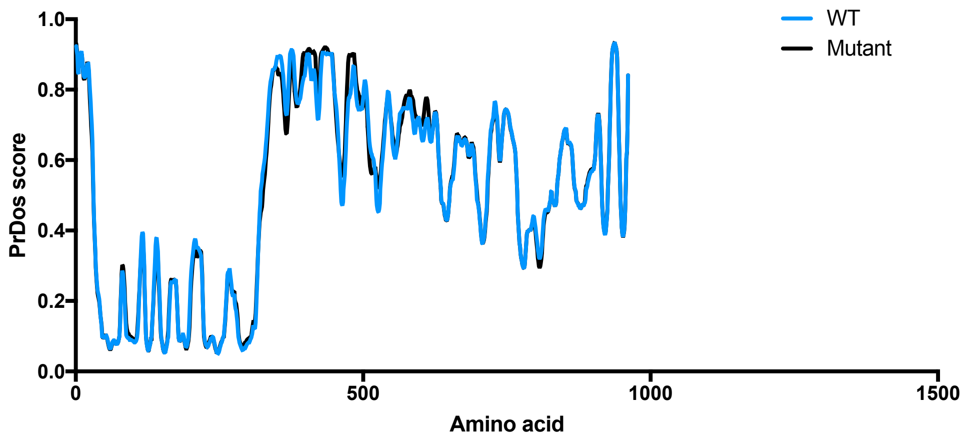

Figure S5: AAK1 WT and mutant display similar structure disorder prediction. Protein Disorder was determined using Protein Disorder Prediction System serve (PrDOS) using the default settings. A scatter plot was generated using the disorder predictions of both the WT and the mutant forms of AAK1.

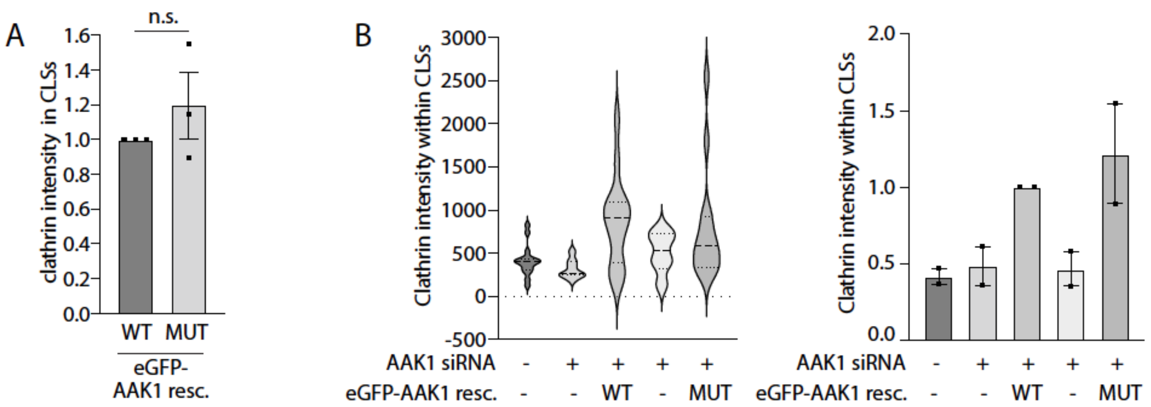

Figure S6. Slight overexpression of wild-type and GlcNAc deficient mutant of AAK1 similarly increase eGFP-clathrin intensity within clathrin labelled structures. ARPE-19 cells were engineered to stably harbor a transgene for inducible expression of eGFP-tagged AAK1 wild-type (WT) or harboring mutations in the 8 predicted O-GlcNAc modification sites (mut). Cells were treated with siRNA to silence endogenous AAK1, or non-targeting siRNA (control). Following siRNA transfection and treatment with dox, cells were fixed and stained to label clathrin heavy chain, followed by imaging by TIRF and epifluorescence microscopy. Experiment is as described in Figure 4D-E, and representative images are shown in Figure 4D. Images were subjected automated detection and analysis of CLSs as described in *Materials and Methods*. (A) Shown are the measurements of clathrin intensity within CLSs comparing only the rescue conditions (with either eGFP-AAK1 wild type and mutant) from *n*=3 independent experiments. (B) Shown are the measurements of clathrin intensity as the distribution of these values in a representative experiment as a violin plot (left panel), or the mean (bar) ± SE from 2 independent experiments, as well as individual values from each experiment (dots).

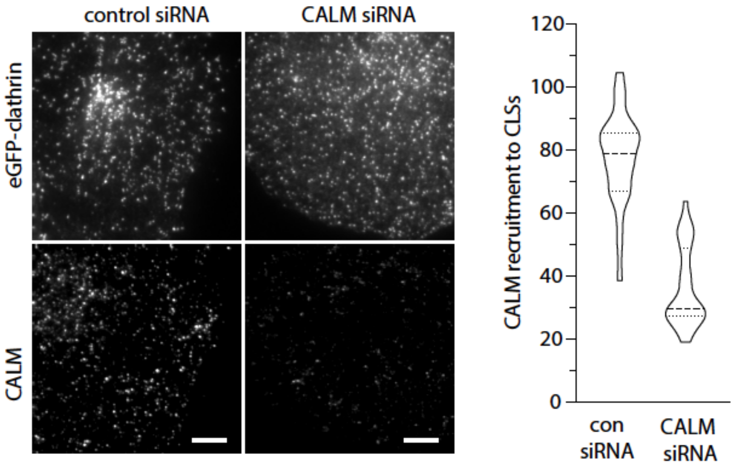

Figure S7. Validation of CALM antibodies for immunofluorescence detection using TIRF microscopy. ARPE-19 cells were treated with siRNA targeting CALM or with non-targeting siRNA (control, con). ARPE-19 cells stably expressing eGFP-clathrin light chain were treated with siRNA to silence CALM or non-targeting (control) siRNA. Following transfection, cells were fixed and stained with anti-CALM antibodies, followed by imaging by TIRF microscopy, with representative images shown in the left panels. Scale 5 µm. CLSs were subjected to automated detection and analysis as described in *Materials and Methods*. Shown are the distributions as a violin plot of measurements of eGFP-clathrin in individual CLSs within a representative experiment. This indicates the specificity of the fluorescence signal in TIRF microscopy derived from the antibodies used to detect endogenous CALM by immunofluorescence staining.

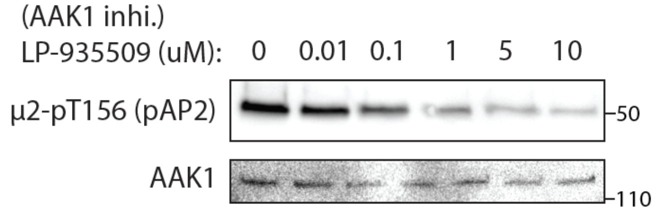

Figure S8: LP-935509 treatment inhibits AP2 phosphorylation. ARPE-19 cells were incubated with different concentrations of LP-935509 as shown for 3 h before lysate preparation. Shown is a representative western blot using whole-cell lysates, showing detection of pAP2 (phospho-Thr156 on the μ2 subunit of AP2) or AAK1. All western blot panels show approximate molecular weight in kDa.
